# CPEB1 drives ferroptosis–neuroinflammation crosstalk in temporal lobe epilepsy via the SIRT1–NRF2 acetylation axis

**DOI:** 10.3389/fimmu.2026.1727784

**Published:** 2026-03-13

**Authors:** Cong Huang, Zhipeng You, Xiaoying Gao, Yunmin He, Shiyi Zhao, Zhijie Fan, Fan Wei, Jiahang Sun

**Affiliations:** 1 Department of Neurosurgery, The Second Affiliated Hospital of Harbin Medical University, Harbin, Heilongjiang, China; 2 Department of Neurosurgery, The First Affiliated Hospital of Harbin Medical University, Harbin, Heilongjiang, China; 3 Department of Anesthesiology, The Fourth Affiliated Hospital of Harbin Medical University, Harbin, Heilongjiang, China

**Keywords:** CPEB1, ferroptosis, neuroinflammation, neuronal injury, SIRT1/NRF2 pathway, temporal lobe epilepsy

## Abstract

**Background:**

Temporal lobe epilepsy (TLE) is a common neurological disorder frequently resistant to pharmacological treatment, yet its molecular mechanisms remain incompletely understood. Cytoplasmic polyadenylation element-binding protein 1 (CPEB1) is a post-transcriptional regulator implicated in neuronal stress responses; however, its role in epilepsy and redox-inflammatory signaling remains unclear.

**Methods:**

An integrative multi-omics strategy combining single-cell transcriptomics, bulk RNA sequencing, and bioinformatics analyses was employed, followed by validation in human epileptic hippocampal tissues, kainic acid (KA)- and pentylenetetrazol (PTZ)-induced mouse models, as well as *in vitro* glutamate-induced neuronal injury models. Mechanistic investigations were performed using adeno-associated virus (AAV)-mediated CPEB1 overexpression and knockdown, together with pharmacological modulation of the SIRT1 and NRF2 pathways.

**Results:**

CPEB1 was markedly upregulated in neurons from both TLE patients and experimental models. Neuronal overexpression of CPEB1 increased seizure susceptibility, exacerbated neuronal loss, and promoted oxidative stress, proinflammatory cytokine release, and ferroptosis, whereas CPEB1 knockdown exerted robust neuroprotective effects. Mechanistically, CPEB1 suppressed SIRT1 activity, leading to enhanced acetylation-dependent destabilization of NRF2, impaired downstream SLC7A11/GPX4 antioxidant signaling, and excessive reactive oxygen species (ROS) accumulation, ultimately triggering ferroptotic neuronal death. Importantly, pharmacological inhibition of SIRT1 or NRF2 abolished the neuroprotective effects of CPEB1 knockdown, confirming the critical role of the CPEB1–SIRT1–NRF2 acetylation axis in TLE pathogenesis.

**Conclusion:**

CPEB1 aggravates neuronal injury in TLE by driving ferroptosis–neuroinflammation crosstalk through suppression of the SIRT1–NRF2 acetylation axis. Targeting this pathway may provide a promising therapeutic strategy for drug-resistant epilepsy and related neurodegenerative disorders.

## 1 Introduction

Epilepsy remains one of the most prevalent chronic neurological disorders globally, with Temporal Lobe Epilepsy (TLE) representing the most common and pharmacoresistant subtype (1). TLE is pathologically characterized by hippocampal sclerosis, aberrant synaptic remodeling, and progressive neuronal loss, yet the molecular mechanisms driving this neurodegeneration remain incompletely defined (2). While traditional research has focused on apoptosis (3) and autophagy (4), recent evidence implicates ferroptosis—an iron-dependent form of regulated cell death driven by lethal lipid peroxidation—as a critical pathogenic mechanism (5). Emerging evidence indicates that ferroptosis plays a pivotal role in neurological disorders such as stroke (6), Parkinson’s disease (7), and Alzheimer’s disease (8), and its potential involvement in epilepsy has garnered increasing attention. For instance, several studies (9) have demonstrated that epileptic seizures elevate Fe²^+^ levels in the brain, promote reactive oxygen species (ROS) accumulation, and suppress antioxidant defense systems—hallmarks of ferroptotic cell death. Moreover, ferroptosis inhibitors such as Ferrostatin-1 have been shown to mitigate neuronal damage and cognitive deficits in animal models of post-traumatic epilepsy (10). Epileptic seizures induce hallmark features of ferroptosis, including glutathione (GSH) depletion, GPX4 inactivation, and mitochondrial shrinkage, suggesting that targeting ferroptotic pathways offers a novel therapeutic strategy to mitigate seizure-induced neuronal injury.

Concurrently, neuroinflammation acts as a pervasive driver of epileptogenesis rather than a mere bystander. Seizures trigger the release of proinflammatory cytokines (IL-1β, IL-6, TNF-α) from activated microglia and astrocytes, which disrupts the blood–brain barrier and exacerbates neuronal excitability (11). Crucially, ferroptosis and neuroinflammation are not isolated events; they are mechanically coupled through oxidative stress, forming a vicious feedback loop that perpetuates brain damage (12–14). However, the upstream molecular “switches” that orchestrate the crosstalk between these two lethal processes at the post-transcriptional level remain largely unknown.

Cytoplasmic polyadenylation element-binding protein 1 (CPEB1) is a core member of the CPEB protein family and contains a highly conserved RNA-binding domain that specifically recognizes cytoplasmic polyadenylation elements (CPEs) within the 3’ untranslated regions of target mRNAs, thereby regulating their polyadenylation and translational elongation (15). CPEB1 is widely expressed throughout the central nervous system and plays a pivotal role in neuronal plasticity, synaptogenesis, and higher-order processes such as learning and memory (16–18). Dysregulated CPEB1 expression has been implicated in a variety of neurological disorders, including Alzheimer’s disease (16), spinal cord injury (19), and schizophrenia (20). In addition, studies in cancer biology (21) have shown that CPEB1 regulates the translation of specific mRNAs involved in oxidative stress responses and iron homeostasis, thereby promoting ferroptosis. Although the roles of CPEB1 in the nervous system have been increasingly recognized, direct evidence of its contribution to epilepsy pathogenesis, particularly TLE, remains lacking. Our previous multi-omics analyses revealed that CPEB1 is significantly upregulated in TLE neurons and is closely associated with the ferroptosis pathway. These findings suggest that CPEB1 may represent a critical regulatory molecule in the pathological progression of TLE, underscoring the need for further investigation into its mechanistic roles and clinical significance.

In this study, by integrating single-cell transcriptomic and bulk RNA sequencing analyses, we identified markedly elevated expression of CPEB1 in neurons of TLE patients and epileptic mouse models, with predominant localization in hippocampal and cortical neurons. Functional assays revealed that CPEB1 overexpression promoted the release of proinflammatory cytokines, amplified neuroinflammatory responses, and aggravated neuronal ferroptosis, whereas CPEB1 knockdown attenuated inflammation, reduced ferroptosis, and conferred significant neuroprotective effects. These findings suggest that CPEB1 acts as a critical molecular nexus linking inflammation and ferroptosis, thereby playing a pivotal role in the pathological mechanisms of TLE. This study elucidates the essential function of CPEB1 in TLE pathogenesis, underscoring its potential as a therapeutic molecular target and providing new insights for the treatment of drug-resistant epilepsy.

## 2 Materials and methods

Detailed experimental protocols, including transcriptome sequencing, t-SNE–based clustering, differential gene expression analysis, GO and KEGG enrichment, RNA and protein structure modeling, molecular docking, and various biochemical assays (ROS, MDA, GSH, Fe²^+^, SOD, LDH, ELISA), are provided in the Supplementary Materials.

### 2.1 Human brain tissue

Brain tissue samples used in this study were obtained from the Department of Neurosurgery at the Second Affiliated Hospital of Harbin Medical University. Written informed consent was obtained from all participants or their legal guardians (n = 12, including TLE patients and controls; 9 males and 3 females; mean age, 55.42 ± 11.89 years). The study protocol was approved by the hospital’s Ethics Committee (Approval No.: YJSKY2024-180), and all experimental procedures were conducted in strict accordance with relevant laws, regulations, and ethical standards.

Prior to surgery, all participants underwent neurological examinations, electroencephalogram (EEG) monitoring, and neuroimaging assessments to localize epileptogenic foci and exclude other brain lesions. Postoperative resected tissues were subjected to pathological examination to confirm histopathological features. Samples in the TLE group were derived from clinically confirmed patients who required surgical resection of epileptogenic lesions. Control samples were collected from non-epileptic patients who underwent craniotomy for benign brain tumors or traumatic brain injury. These control patients had no history of epileptic seizures, no evidence of epileptiform discharges, and were matched to the TLE group in terms of age and sex distribution.

All surgically resected tissues were immediately processed: a portion was snap-frozen for molecular analyses (including RNA extraction and protein detection), while another portion was preserved for immunohistochemical evaluation. To ensure accuracy and scientific rigor, all procedures adhered to standardized protocols and principles of patient privacy protection. No financial compensation was provided to participants. Detailed clinical and pathological characteristics of the study samples are presented in **Table 1**.

**Table 1:** Clinical information of the human subjects in this study.

Number	Patient	Sex	Age	Histology	Sample localization	Sample type	Experiments
1	TLE1	M	65	TLE, Left hippocampal sclerosis	neocortex, hippocampus	fresh sample	WB
2	TLE2	M	31	TLE, Right hippocampal sclerosis	neocortex, hippocampus	fresh sample	WB
3	TLE3	M	46	TLE, Left hippocampal sclerosis	neocortex, hippocampus	fresh sample	WB
4	TLE4	M	56	TLE, Left hippocampal sclerosis	neocortex, hippocampus	fresh sample	WB
5	TLE5	F	68	TLE, Left hippocampal sclerosis	neocortex, hippocampus	fresh sample	WB, IF
6	TLE6	M	43	TLE, Left hippocampal sclerosis	neocortex, hippocampus	fresh sample	WB, IF
7	Normal1	M	51	The right basal ganglia hemorrhage	neocortex	fresh sample	WB, IF
8	Normal2	F	48	Intracranial hematoma	neocortex	fresh sample	WB
9	Normal3	M	70	Intracranial hematoma	neocortex	fresh sample	WB
10	Normal4	F	67	Intracranial hematoma	neocortex	fresh sample	WB
11	Normal5	M	59	The right basal ganglia hemorrhage	neocortex	fresh sample	WB
12	Normal6	M	61	Intracranial hematoma	neocortex	fresh sample	WB

### 2.2 Animals

Male C57BL/6J mice (6–8 weeks old, weighing 20–25 g) were used in this study. All animals were obtained from the Animal Experimental Center of the Second Affiliated Hospital of Harbin Medical University. Based on preliminary variance estimates and power analysis (α = 0.05, power = 0.8), a minimum of 6 animals per group was required to detect biologically meaningful differences. To ensure sufficient statistical power while accounting for potential model failure or mortality, we included 10–12 mice per group in the experimental design, resulting in a total of approximately 160 mice. For specific biochemical or histological analyses, 4–8 biological replicates were randomly selected per group depending on tissue availability and technical feasibility. Mice were housed in an individually ventilated cage (IVC) system under controlled temperature and humidity conditions, with a 12-hour light/dark cycle, and were provided ad libitum access to standard chow and water.

All animal experiments were conducted in strict accordance with the Regulations on the Administration of Laboratory Animals and relevant ethical guidelines. The experimental protocol was reviewed and approved by the Animal Ethics Committee of the Second Affiliated Hospital of Harbin Medical University (Approval No.: YJSDW2024-266). The principles of the 3Rs (Replacement, Reduction, and Refinement) were rigorously followed to minimize animal use and suffering while ensuring scientific integrity.

For all surgical procedures, including epilepsy model induction, mice were anesthetized with tribromoethanol (250 mg/kg, intraperitoneal injection). The depth of anesthesia was confirmed by the absence of pedal withdrawal and corneal reflexes before initiating any surgical manipulation.

At the end of the experiments, prior to tissue collection, animals were re-anesthetized with the same agent and dose, and euthanasia was performed by intraperitoneal administration of an overdose of tribromoethanol (≥400 mg/kg) while the animals remained under deep anesthesia. All tribromoethanol solutions were freshly prepared and filtered before use to ensure stability and minimize tissue irritation. All procedures complied with the AVMA Guidelines for the Euthanasia of Animals (2020) and institutional policies for the care and use of laboratory animals.

### 2.3 Kainic acid model

In this study, a mouse model of temporal lobe epilepsy (TLE) was established using kainic acid (KA) induction. Prior to experimentation, mice were anesthetized via intraperitoneal injection of 4% sodium pentobarbital and secured in a stereotaxic apparatus. A microsyringe was then used to bilaterally inject 0.6 µL of KA solution (0.5 µg/µL, Sigma, USA) into the hippocampal CA1 region. Injection coordinates were determined relative to the bregma: anteroposterior (AP) = –2.0 mm, mediolateral (ML) = ± 1.5 mm, and dorsoventral (DV) = –2.0 mm (22). Control mice received an equivalent volume of sterile saline. To ensure adequate diffusion, the needle was retained in place for 5 minutes before being slowly withdrawn.

Following induction, seizure behaviors were assessed using the Racine scale (23), with mice reaching grade IV or higher considered successfully modeled. Twenty-eight days post-induction, brain tissues were harvested for histological, molecular, and biochemical analyses to evaluate CPEB1 expression and its biological functions in the epilepsy model.

### 2.4 Pentylenetetrazol model

To establish a chronic epilepsy model, seizures were induced in mice through repeated intraperitoneal injections of pentylenetetrazol (PTZ). A sub-convulsive dose of 35 mg/kg was administered every 48 hours for a total of 14 injections. Following each injection, mice were continuously monitored for 30 minutes, and seizure severity was assessed using the Racine scale. Parameters recorded included seizure scores, latency to the first generalized tonic–clonic seizure (GTC), and GTC duration. This protocol reliably reproduces the chronic progression of epilepsy and enables systematic evaluation of the role of CPEB1 in regulating seizure susceptibility. After the 14th injection, mice were euthanized, and brain tissues were harvested for molecular and morphological analyses.

### 2.5 Adeno-associated virus injection

The CPEB1-related adeno-associated viruses (AAVs) used in this study were provided by Obio Technology (Shanghai, China) Corp., Ltd. The overexpression vector was pcAAV-CMV-Cpeb1-linker-EGFP-3xFLAG-WPRE, with a titer of 2.73 × 10¹² v.g./mL, while the knockdown vector was pAAV-U6-shRNA(Cpeb1)-CMV-EGFP-WPRE, with a titer of 8.1 × 10¹² v.g./mL.

Under anesthesia, mice were positioned in a stereotaxic apparatus, and viral solutions were bilaterally injected into the hippocampal CA1 regions using a 1.0 μL microsyringe. Injection coordinates relative to the bregma were as follows: anteroposterior (AP) = –2.0 mm, mediolateral (ML) = ± 1.5 mm, and dorsoventral (DV) = –1.7 mm. Each side received 0.5 μL at a controlled rate of 0.2 μL/min. To ensure sufficient viral diffusion, the needle was kept in place for 5 minutes before withdrawal.

Three weeks after viral delivery, mice were subjected to KA- or PTZ-induced epilepsy models to assess the functional role of CPEB1 regulation in seizure activity and neuronal ferroptosis.

### 2.6 Western blotting

In this study, Western blotting was performed to assess protein expression levels in tissue samples. Total hippocampal proteins were extracted from mice using RIPA lysis buffer (Beyotime, China) supplemented with protease inhibitors, following the manufacturer’s protocol. Protein concentrations were determined, and equal amounts of protein were separated by SDS-PAGE and subsequently transferred onto polyvinylidene fluoride (PVDF) membranes (Millipore, USA). After transfer, membranes were blocked with 5% non-fat milk at room temperature for 2 hours to minimize nonspecific binding. Specific primary antibodies (detailed in **Table 2**) were then applied and incubated overnight at 4 °C. The following day, membranes were washed three times with TBST buffer and incubated with HRP-conjugated secondary antibodies at room temperature for 2 hours. Protein bands were visualized using an ECL chemiluminescence detection kit (Epizyme, China) and imaged with a chemiluminescence detection system. Relative protein expression levels were quantified through grayscale analysis software, with GAPDH serving as the internal control for normalization.

**Table 2:** Antibody details.

Antibody	Source	Catalog number	Species	Applications
CPEB1	Proteintech	13274-1-AP	Rabbit	WB (1:1000) IF (1:100) RIP (10μg)
GAPDH	Proteintech	10494-1-AP	Rabbit	WB (1:5000)
NeuN	Proteintech	66836-1-lg	Mouse	IF (1:100)
GFAP	Proteintech	60190-1-lg	Mouse	IF (1:100)
Iba-1	Proteintech	10904-1-AP	Rabbit	IF (1:200)
SLC7A11	Abmart	T57046S	Rabbit	WB (1:1000)
GPX4	Proteintech	67763-1-lg	Mouse	WB (1:2000)
BAP-1	Proteintech	10398-1-AP	Rabbit	WB (1:1000)
P53	Proteintech	10442-1-AP	Rabbit	WB (1:10000)
NRF2	Proteintech	80593-1-RR	Rabbit	WB (1:2500)
AC-NRF2 (Lys599)	Uptbio	PLN000063	Rabbit	WB (1:1000)
SIRT1	Proteintech	13161-1-AP	Rabbit	WB (1:3000)

### 2.7 Quantitative real-time PCR

Total RNA from mouse hippocampal tissues was isolated using the TRIzol reagent (Invitrogen, USA) following the manufacturer’s protocol. RNA concentration and purity were assessed using a Nanodrop spectrophotometer. Complementary DNA (cDNA) was synthesized from 1 μg of total RNA using the TransScript^®^ One-Step cDNA Synthesis Kit (TransGen Biotech, China) according to the manufacturer’s instructions. Quantitative real-time PCR (qPCR) was performed using the PerfectStart^®^ Green qPCR SuperMix kit (TransGen Biotech, China) on a CFX96 Real-Time PCR System (Bio-Rad, USA). Relative gene expression was calculated using the 2^-ΔΔCt method, with GAPDH serving as the internal normalization control. Primer sequences are listed in **Table 3**.

**Table 3:** Primer sequences used for qRT-PCR.

Gene target	Species	Forward primer (5'-3')	Reverse primer (5'-3')
NFE2L2 (NRF2)	Mouse	TCTTGGAGTAAGTCGAGAAGTGT	GTTGAAACTGAGCGAAAAAGGC
SLC7A11	Mouse	GGCACCGTCATCGGATCAG	TCCCAAGAGCCAAAGTGCCA
GPX4	Mouse	GAGGCAAGACCGAAGTAAACTAC	CCGAACTGGTTACACGGGAA
SIRT1	Mouse	GATACGTTGGCACCGAGTG	TGCCATCTCCAGTGTTCTTG
GAPDH	Mouse	AGGTCGGTGTGAACGGATTTG	GGGGTCGTTGATGGCAACA

### 2.8 RNA immunoprecipitation assay

To validate the direct interaction between CPEB1 protein and SIRT1 mRNA, RIP assays were performed using the Magna RIP™ RNA-Binding Protein Immunoprecipitation Kit (Millipore, USA) according to the manufacturer’s instructions. Briefly, hippocampal and cortical tissues were lysed in RIP lysis buffer containing protease and RNase inhibitors. The lysates were immunoprecipitated with magnetic beads conjugated to an anti-CPEB1 antibody (Abcam) or normal rabbit IgG (negative control) at 4 °C overnight. After washing, proteinase K was added to digest proteins, and the immunoprecipitated RNA was purified using phenol-chloroform extraction. The enrichment of SIRT1 mRNA in the immunoprecipitates was quantified by qRT-PCR using specific primers. The results were normalized to the Input fraction and expressed as fold enrichment relative to the IgG control. Relevant antibody information is listed in **Table 2**, and primer sequences are provided in **Table 3**.

### 2.9 Immunofluorescence staining

To examine the expression and cellular localization of target proteins in brain tissues, immunofluorescence staining was performed on paraffin-embedded sections. Briefly, tissue sections were deparaffinized and immersed in 1× citrate buffer, followed by antigen retrieval using a high-pressure heat treatment at high temperature for 10 minutes. The sections were then incubated in 0.3% Triton X-100 solution (Beyotime, China) at room temperature for 15 minutes to enhance membrane permeability. To block nonspecific antibody binding, 10% goat serum (Boster, China) was applied at 37 °C for 30 minutes. After blocking, sections were incubated overnight at 4 °C with specific primary antibodies (listed in **Table 2**). The next day, sections were thoroughly washed with PBS and incubated with fluorescently conjugated secondary antibodies for 1 hour at room temperature in the dark. Finally, nuclei were counterstained with DAPI solution (Beyotime, China), and fluorescence images were captured and analyzed using a microscope (Nikon, Japan).

### 2.10 Drug administration

To further validate the molecular mechanisms by which CPEB1 regulates neuroinflammation and ferroptosis, pharmacological interventions were conducted in CPEB1 knockdown mice using the NRF2 inhibitor ML385 and the SIRT1 inhibitor EX-527. ML385 (MCE, USA) was dissolved in PBS containing 5% dimethyl sulfoxide (DMSO) and administered intraperitoneally at a dose of 30 mg/kg (24). This dosage was selected based on prior pharmacokinetic studies in mice, which reported a plasma half-life (t_1_/_2_) of approximately 2.8 hours and demonstrated effective blood–brain barrier penetration sufficient to inhibit NRF2-mediated transcriptional activity in the brain (25, 26). Control mice received an equivalent volume of vehicle solution (5% DMSO in PBS). Drug administration commenced prior to KA model induction, given once daily for three consecutive days, and continued after KA injection for a total of four days to ensure adequate steady-state drug levels during the acute phase of excitotoxicity (27).

Similarly, EX-527 (MCE, USA), a highly selective SIRT1 inhibitor (IC50 = 38 nM), was dissolved in PBS containing 5% DMSO and administered intraperitoneally at a dose of 10 mg/kg. This dosing regimen is well-established to achieve pharmacologically active concentrations in the central nervous system, given its lipophilic nature and ability to cross the blood-brain barrier (28). The administration schedule was identical to that of ML385, consisting of once-daily injections prior to KA induction and continued dosing after KA injection until the animals were sacrificed (29). All drugs were freshly prepared immediately before use under light-protected conditions. Dosage and administration frequency were determined based on published literature and adjusted according to body weight to ensure both efficacy and safety of the intervention.

### 2.11 Cell culture

To investigate the mechanisms by which CPEB1 contributes to epilepsy-associated neuronal injury, we established a glutamate-induced excitotoxicity model to mimic *in vitro* neuronal damage under epileptic conditions (30, 31). The immortalized mouse hippocampal neuronal cell line HT22 was obtained from Procell Life Science & Technology Co., Ltd. (Wuhan, China; Catalog No.: CL-0697). Cells were maintained in Dulbecco’s Modified Eagle Medium (DMEM, Sigma, USA; Catalog No.: D5796) supplemented with 10% fetal bovine serum (FBS), 100 U/mL penicillin, and 100 μg/mL streptomycin, and cultured in a humidified incubator at 37 °C with 5% CO_2_. Prior to experimentation, cells were seeded at appropriate densities into 6-well or 96-well plates and allowed to adhere for 24 hours before treatment. Excitotoxicity was induced by adding 5 mM glutamate (Glu) to the culture medium, thereby replicating the neuronal injury environment associated with epileptic seizures and providing an *in vitro* platform for investigating the functions and mechanisms of CPEB1.

### 2.12 Transfection experiment

To assess the role of CPEB1 in regulating ferroptosis-associated mechanisms in neuronal cells, transient transfection of HT22 cells was performed using Lipofectamine™ 8000 reagent (Beyotime, China). Cells were transfected with either a CPEB1 expression plasmid [CPEB1-pcDNA3.1(+)] or an empty vector control [pcDNA3.1(+)], strictly following the manufacturer’s instructions. Six hours post-transfection, the medium was replaced with fresh complete medium, and cells were further cultured for 48 hours to ensure sufficient expression and functional activity of the target gene. Subsequently, glutamate (5 mM) and the protein synthesis inhibitor cycloheximide (CHX, 15 μmol/L; MCE, USA) were added to the culture system. After 24 hours of treatment, cells were harvested for protein extraction and subsequent analyses (32). This experiment was designed to determine whether CPEB1 overexpression promotes neuronal ferroptosis by regulating protein stability and thereby modulating NRF2 and its downstream effectors.

### 2.13 Nissl staining

To evaluate the effects of CPEB1 on neuronal survival in epilepsy models, Nissl staining was performed for histological analysis of mouse brain tissues, following standard protocols described in previous studies (33). Mice were anesthetized using conventional procedures and subjected to cardiac perfusion with phosphate-buffered saline (PBS), followed by fixation with 4% paraformaldehyde. Brain tissues were subsequently dehydrated, embedded, and sectioned into continuous slices of 10 μm thickness. The sections were incubated in cresyl violet staining solution (Beyotime, China; Catalog No.: C0117) at room temperature for 10 minutes. After staining, dehydration, clearing, and mounting were conducted according to routine protocols. Images were then captured and analyzed using a Nikon optical microscope (Nikon, Japan). Staining results were used to assess neuronal morphological integrity and changes in neuronal density across different hippocampal regions (including CA1 and CA3), thereby determining the impact of CPEB1 regulation on epilepsy-associated neuronal injury.

### 2.14 Transmission electron microscopy

To investigate ultrastructural changes in neuronal mitochondria during ferroptosis, transmission electron microscopy (TEM) was employed for morphological analysis of mouse brain tissues. Hippocampal or cortical tissues were first fixed in 2.5% glutaraldehyde at 4 °C overnight. The tissues were then dehydrated through a graded ethanol series and embedded in epoxy resin according to standard procedures. Ultrathin sections were prepared using an ultramicrotome and mounted onto 200-mesh copper grids. Sections were sequentially stained with 2% uranyl acetate and 0.04% lead citrate to enhance organelle contrast. Mitochondrial ultrastructure was subsequently examined, and images were captured using a Hitachi transmission electron microscope. This experiment was designed to evaluate the effects of CPEB1 regulation on characteristic ferroptotic mitochondrial morphology in epileptic neurons—including mitochondrial shrinkage, cristae reduction, and increased membrane density—thereby providing morphological evidence for the occurrence of ferroptosis.

### 2.15 Enzyme-linked immunosorbent assay

After dissection, cortical and hippocampal tissues were immediately isolated from mouse brains under ice-bath conditions. The tissues were homogenized in PBS buffer and centrifuged at 5000 rpm for 15 minutes at 4 °C, after which the supernatants were collected. The concentrations of interleukin-1β (IL-1β), interleukin-6 (IL-6), and tumor necrosis factor-α (TNF-α) in cortical and hippocampal tissues were quantified using commercial ELISA kits (Solarbio, Beijing, China; Catalog Nos.: SEKM-0002, SEKM-0007, SEKM-0034), strictly according to the manufacturer’s instructions (34).

### 2.16 Statistical analysis

All experimental data were analyzed using GraphPad Prism 9.0 software, and results are presented as mean ± standard deviation (Mean ± SD). Normality of the data distribution was assessed using the Shapiro-Wilk test, and homogeneity of variance was verified using Levene’s test. Comparisons between two groups were conducted using Student’s t-test, while one-way ANOVA or two-way ANOVA was applied for multiple-group comparisons according to the experimental design, followed by Tukey’s multiple comparison test to evaluate intergroup differences. Statistical significance was defined as p < 0.05, with notations as follows: ns, not significant (p ≥ 0.05); *p < 0.05; **p < 0.01; ***p < 0.001; ****p < 0.0001. All statistical analyses were performed under the assumptions of normality and homogeneity of variance to ensure the rigor and reliability of the results.

## 3 Result

### 3.1 Single-cell transcriptomics reveals differentially expressed genes and functional reprogramming in neurons of TLE

**Figure 1:**
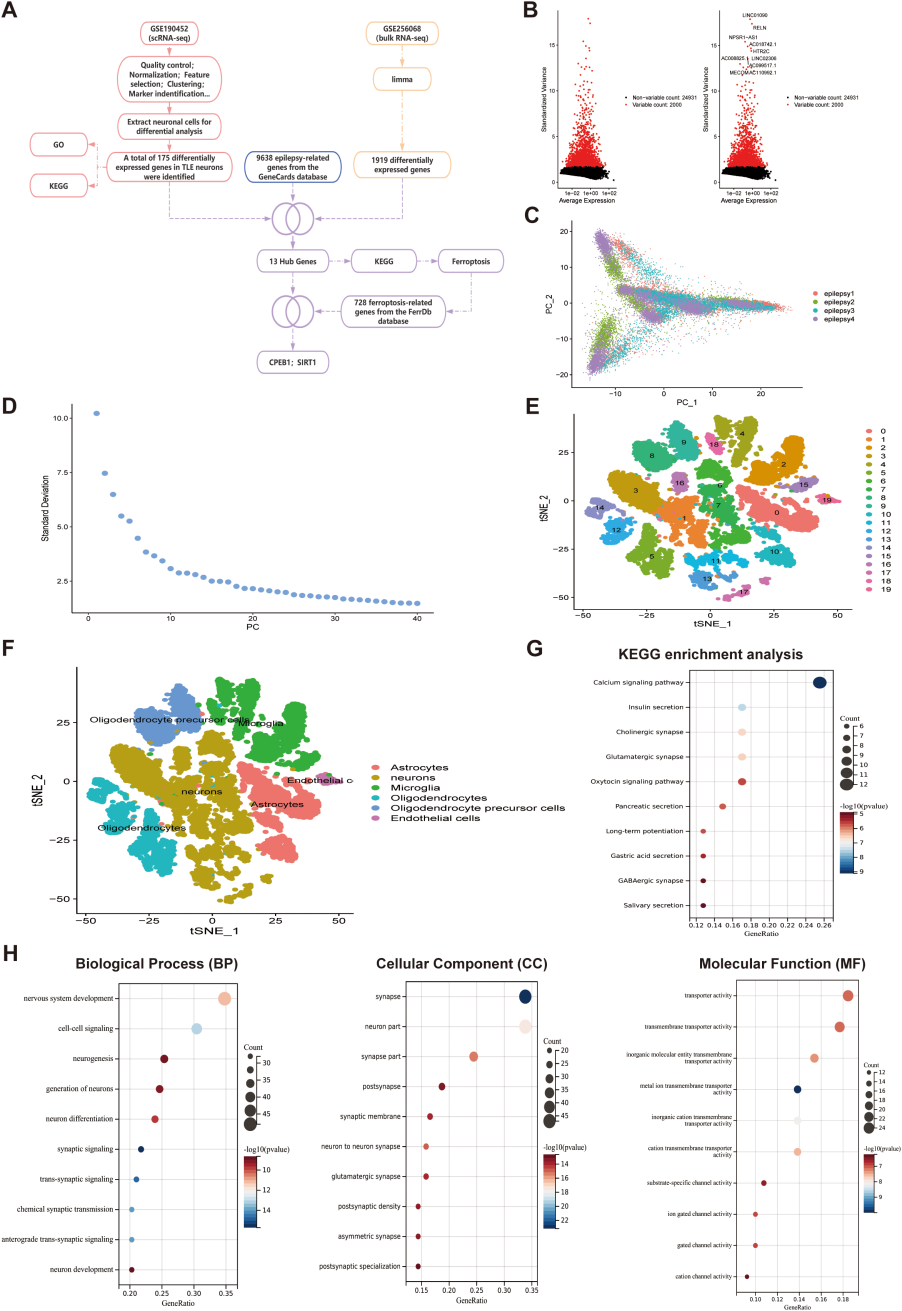
scRNA-seq cell clustering and annotation. **(A)** Schematic overview of the integrative analytical strategy combining single-cell RNA sequencing, bulk RNA sequencing, epilepsy-related gene annotation, and ferroptosis database intersection, leading to the identification of CPEB1 and the proposed SIRT1–NRF2 regulatory axis. **(B)** Differential gene expression analysis identified highly variable genes, with red representing the top 2000 highly variable genes and black representing low-variable genes; the top ten genes ranked by expression among the highly variable genes are labeled. **(C)** Distribution of cells along PC1 and PC2 dimensions, with each dot representing a single cell. **(D)** Standard deviation distribution of principal components, where larger standard deviations indicate greater importance of the corresponding principal component. **(E)** t-SNE clustering visualization, with different colors representing distinct cell clusters, showing cellular aggregation and distribution patterns. **(F)** Cell annotation results based on t-SNE clustering, with each color representing a cell subpopulation. **(G)** KEGG pathway enrichment analysis of neuronal differentially expressed genes, where the x-axis represents the GeneRatio and the y-axis represents KEGG terms; circle size indicates the number of enriched genes, and color indicates enrichment *p*-value. **(H)** GO functional analysis of neuronal differentially expressed genes, including biological process (BP), cellular component (CC), and molecular function (MF); the x-axis represents the GeneRatio and the y-axis represents GO terms, with circle size indicating the number of enriched genes and color indicating enrichment *p*-value.

To identify key molecular drivers of neuronal injury in TLE, we performed an integrated multi-omics analysis combining single-cell RNA sequencing, bulk RNA sequencing, epilepsy-related gene annotation, and ferroptosis databases. The overall analytical workflow and hypothesis-generation strategy are summarized in **Figures 1A**, which illustrates the stepwise identification of CPEB1 and the proposed SIRT1–NRF2 regulatory axis.

In this study, the single-cell transcriptome dataset GSE190452 from TLE patients was obtained from the GEO database, comprising four human hippocampal tissue samples. Data integration and quality control were performed using the Seurat package. The quality control thresholds were defined as follows: number of detected genes per cell (nFeature_RNA) > 500, total transcript counts (nCount_RNA) between 1000 and 20000, and mitochondrial gene percentage (percent.mt) < 10% (**Supplementary Figure 1A**). Cells meeting these criteria were retained for subsequent analyses, whereas low-quality cells were excluded.

Correlation analysis of sequencing depth confirmed that the retained cells exhibited consistent and reliable expression profiles, ensuring high-quality data (**Supplementary Figure 1B**). The top 2000 highly variable genes were then selected for downstream dimensionality reduction and clustering analyses (**Figure 1B**). Principal component analysis (PCA) was performed for initial dimensionality reduction, followed by visualization of the top principal component gene expression patterns (PC1–PC4; **Supplementary Figure 1C**) and a two-dimensional distribution of cells along PC1 and PC2 (**Figure 1C**), both of which demonstrated no significant batch effects. Based on the ElbowPlot (**Figure 1D**), the top 20 principal components were selected for clustering. Subsequently, the t-SNE algorithm was applied for nonlinear dimensionality reduction, identifying 20 distinct cell clusters (**Figure 1E**), from which marker genes were extracted (**Supplementary Figure 1D**). Cell type annotation was performed using the singleR package in combination with the CellMarker database, ultimately identifying six major cell types: neurons, microglia, oligodendrocytes, astrocytes, endothelial cells, and oligodendrocyte precursor cells (**Figure 1F**).

Focusing on neurons, 175 differentially expressed genes (DEGs) were identified in TLE neurons. KEGG pathway enrichment analysis revealed significant enrichment in neurotransmitter-associated pathways, including calcium signaling, cholinergic synapse, glutamatergic synapse, and GABAergic synapse (**Figure 1G**). GO functional enrichment analysis further demonstrated that, under “Biological Process” (BP), DEGs were involved in neuronal development, neurogenesis, and synaptic signaling; under “Cellular Component” (CC), they were enriched in neuronal and synaptic structures; and under “Molecular Function” (MF), they were predominantly associated with ion channel activity and transmembrane ion transport (**Figure 1H**).

In summary, single-cell transcriptomic analysis revealed profound gene expression reprogramming in TLE neurons. These DEGs may play central roles in TLE pathogenesis, providing a foundation for identifying key functional molecules and potential therapeutic targets.

### 3.2 Multi-omics integrative analysis identifies CPEB1 as a key candidate gene involved in ferroptosis

**Figure 2:**
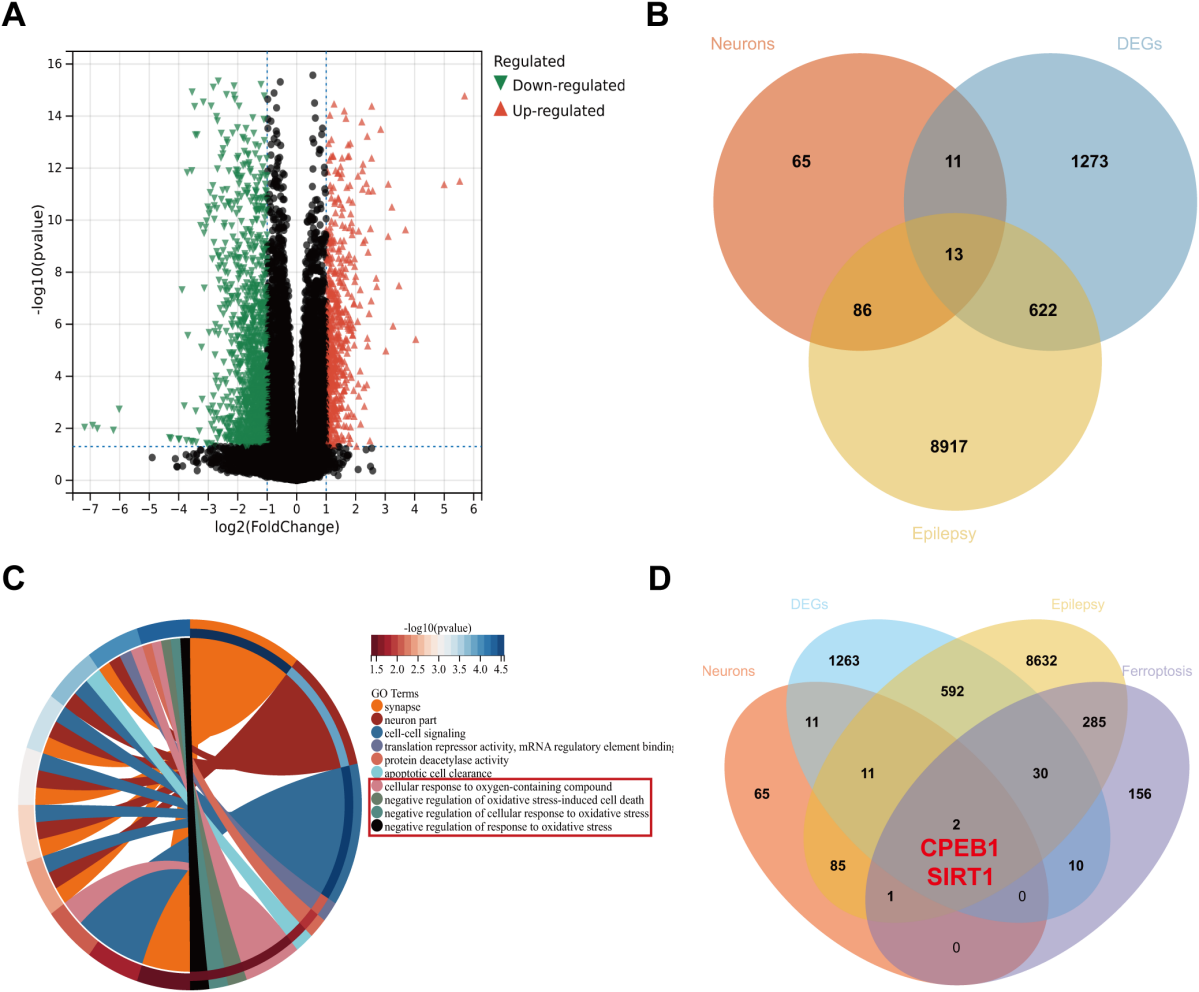
Identification of target genes in TLE. **(A)** Volcano plot of differentially expressed genes in the transcriptome (x-axis: log2(FoldChange), y-axis: -log10(pvalue); green triangles indicate downregulated genes, red triangles indicate upregulated genes, and black dots indicate non-significant genes; control group n=11, TLE group n=59). **(B)** Venn diagram showing the intersection of differentially expressed genes (DEGs), epilepsy-related genes from the GeneCards database, and neuronal DEGs in TLE. **(C)** Circular GO enrichment plot of the 13 core DEGs highly associated with TLE neurons, showing the top 10 GO terms. **(D)** Venn diagram showing the intersection of DEGs, epilepsy-related genes from the GeneCards database, neuronal DEGs in TLE, and ferroptosis-related genes.

To further investigate the potential molecular mechanisms underlying TLE, we analyzed the bulk RNA-seq dataset GSE256068 from the GEO database. Differential expression analysis identified 1919 differentially expressed genes (DEGs) (**Figure 2A**). To screen for genes most closely associated with epilepsy, these DEGs were intersected with 9638 epilepsy-related genes from the GeneCards database and the 175 neuron-specific DEGs identified in our single-cell analysis (**Figure 2B**), yielding 13 core DEGs strongly associated with TLE neurons (**Table 4**). GO functional enrichment analysis revealed that these 13 genes were predominantly involved in key biological processes, including neuronal structural organization, protein deacetylation, and cellular oxidative stress responses (**Figure 2C**), suggesting their potential contribution to TLE pathophysiology.

**Table 4:** Thirteen core DEGs highly associated with TLE neurons.

Gene	log_2_(fold change)	pvalue	Type
NRG1	-1.07014328	0.0295358558553416	Down
DLGAP2	1.39625763355877	4.81491873403764e-06	Up
CPEB1	1.51803402251251	0.00150661047659342	Up
CBLN2	-1.499203308	0.00101845981698571	Down
VSNL1	-1.205229639	4.26006494466196e-05	Down
CUX2	-1.518774504	0.0227270324913663	Down
MERTK	-1.403572482	0.0345087529205321	Down
SLC9A9	1.18362855601696	0.000140659864927933	Up
ATP1A2	-1.172629379	0.000855754877977101	Down
DISC1	-1.726324496	5.18177359361067e-06	Down
ADARB2	-3.528476247	0.0330073424252848	Down
GLIS3	-1.037313395	0.000840049460471197	Down
SIRT1	-2.707068209	0.00279426879221806	Down

To further identify candidate molecules implicated in ferroptosis, the 13 core genes were intersected with 728 ferroptosis-related genes from the FerrDb database (**Figure 2D**), ultimately highlighting two potential key regulators: CPEB1 and SIRT1. Previous studies have reported that CPEB1 participates in ferroptosis in pancreatic and gastric cancers (21, 35), yet its role and mechanisms in neuronal ferroptosis within the context of TLE remain poorly understood. Elucidating the function of CPEB1 may provide critical insights into TLE pathogenesis and establish a foundation for novel therapeutic strategies. Therefore, CPEB1 was designated as the central focus of this study, with the aim of determining whether it contributes to TLE-associated neuronal injury through regulation of the ferroptosis pathway and clarifying its mechanistic role in disease progression.

### 3.3 Expression and distribution of CPEB1 in the TLE brain

**Figure 3:**
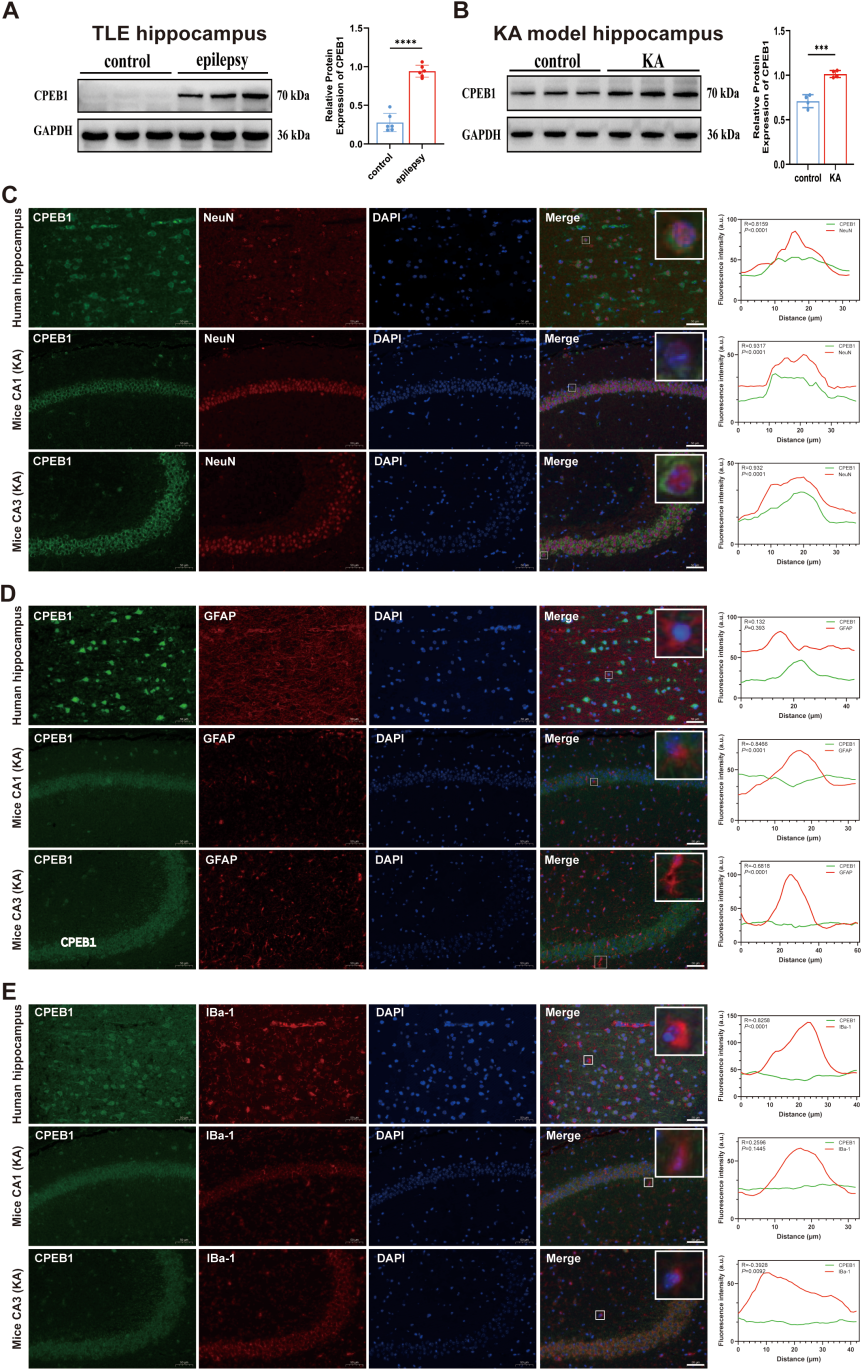
Expression and cellular localization of CPEB1 in the hippocampus of TLE patients and KA-induced epileptic mice. **(A)** Representative Western blot images and quantitative analysis showing CPEB1 protein expression in the hippocampus of temporal lobe epilepsy (TLE) patients and non-epileptic control subjects. GAPDH was used as a loading control. **(B)** Representative Western blot images and quantitative analysis showing CPEB1 protein expression in the hippocampus of kainic acid (KA)-induced epileptic mice and control mice. **(C)** Immunofluorescence staining of CPEB1 (green), NeuN (neuronal marker, red), and DAPI (blue) in the hippocampus of TLE patients and in the CA1 and CA3 regions of KA-induced epileptic mice. Line-scan analyses show fluorescence intensity profiles indicating colocalization of CPEB1 with NeuN. **(D)** Immunofluorescence staining of CPEB1 (green), GFAP (astrocyte marker, red), and DAPI (blue) in the hippocampus of TLE patients and in the CA1 and CA3 regions of KA-induced epileptic mice. Line-scan analyses indicate minimal colocalization between CPEB1 and GFAP. **(E)** Immunofluorescence staining of CPEB1 (green), Iba-1 (microglial marker, red), and DAPI (blue) in the hippocampus of TLE patients and in the CA1 and CA3 regions of KA-induced epileptic mice. Line-scan analyses indicate minimal colocalization between CPEB1 and Iba-1. Scale bar = 50 μm. Data are presented as mean ± SEM. ***P < 0.001, ****P < 0.0001.

To validate the expression profile of CPEB1 in TLE, we first examined its protein levels in hippocampal and cortical tissues from TLE patients and compared them with non-epileptic controls. Given that TLE primarily originates from the hippocampus but progressively involves extrahippocampal regions, including the cortex, during chronic seizure propagation and network remodeling (36, 37), both regions were systematically analyzed to determine whether CPEB1 dysregulation represents a focal or network-wide pathological feature. Western blot analysis revealed that CPEB1 protein levels were significantly elevated in both the hippocampus and cortex of TLE patients (**Figure 3A**; **Supplementary Figure 2A**). We next assessed CPEB1 expression in experimental models of chronic epilepsy. In the kainic acid (KA)-induced model, which recapitulates key pathological features of hippocampal sclerosis, CPEB1 expression was markedly increased in both hippocampal and cortical tissues (**Figure 3B**; **Supplementary Figure 2B**). Similarly, in the pentylenetetrazol (PTZ)-kindling model, Western blot analyses demonstrated robust upregulation of CPEB1 in both regions (**Supplementary Figures 3A, B**). These findings indicate that CPEB1 elevation is a reproducible and model-independent molecular alteration across distinct epilepsy paradigms.

Although bioinformatics analyses predicted that CPEB1 is predominantly expressed in neurons, its precise cellular localization in epileptic brain tissue required experimental validation. High-resolution immunofluorescence staining was therefore performed in hippocampal sections from TLE patients, KA-induced epileptic mice, and PTZ-kindled mice. In all conditions examined, CPEB1 exhibited prominent colocalization with the neuronal marker NeuN and was primarily localized within neuronal soma (**Figure 3C**; **Supplementary Figures 2C**, **3C**). Quantitative analyses demonstrated that the majority of CPEB1-positive cells were NeuN-positive neurons, and fluorescence intensity profiling and Pearson’s correlation analyses confirmed a high degree of spatial overlap between CPEB1 and NeuN signals.

To determine whether CPEB1 is also expressed in non-neuronal cell types, double immunofluorescence staining was performed with astrocytic (GFAP) and microglial (Iba1) markers. In both human TLE samples and epileptic mouse models, CPEB1 showed minimal colocalization with GFAP-positive astrocytes or Iba1-positive microglia in the hippocampus (**Figures 3D, E**), as well as in the cortex (**Supplementary Figures 2D, E**, **3D, E**). Quantitative colocalization analyses revealed low correlation coefficients and limited signal overlap, indicating that neuronal enrichment represents the predominant pattern of CPEB1 upregulation in epileptic brain tissue.

Taken together, these results demonstrate that CPEB1 is consistently upregulated in both hippocampal and cortical regions across human TLE samples and multiple experimental epilepsy models, with predominant localization in neurons. Given the central role of the hippocampus as the primary epileptogenic focus in TLE, subsequent mechanistic and functional analyses were therefore focused on hippocampal neurons, while cortical data are presented to support the network-wide relevance of CPEB1 dysregulation.

### 3.4 CPEB1 increases seizure susceptibility and neuronal death following status epilepticus

**Figure 4:**
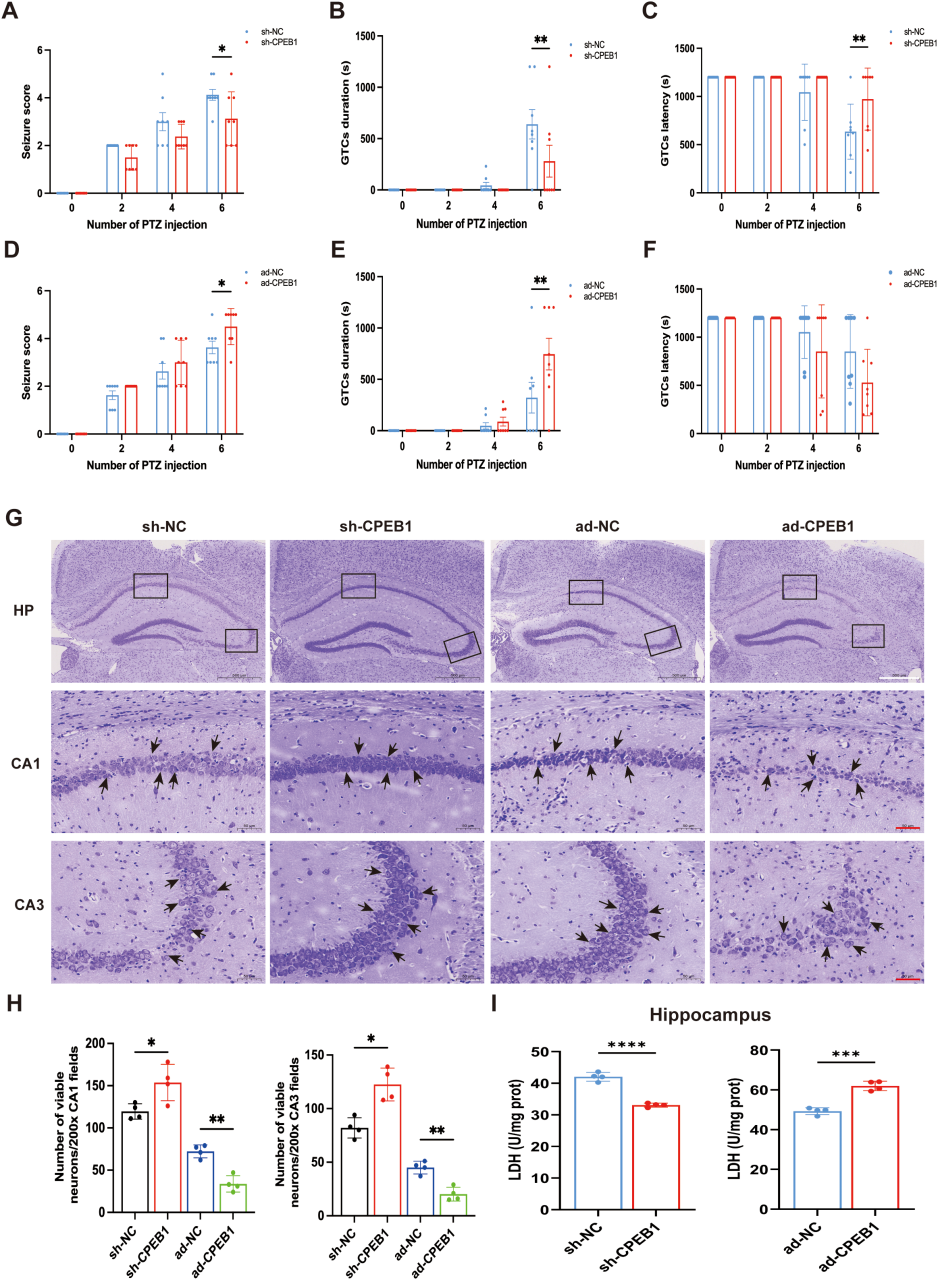
CPEB1 increases seizure susceptibility and neuronal death following status epilepticus. **(A)** Seizure scores, **(B)** duration of generalized tonic-clonic seizures (GTCs), and **(C)** latency to GTCs, used to evaluate the effect of CPEB1 knockdown on seizure susceptibility in mouse epilepsy models. **(D)** Seizure scores, **(E)** duration of GTCs, and **(F)** latency to GTCs, used to evaluate the effect of CPEB1 overexpression on seizure susceptibility in mouse epilepsy models. **(G)** Representative Nissl staining images showing the effect of CPEB1 on surviving neurons in KA-treated mice. Arrows indicate Nissl-positive cells; white scale bar = 500 µm, red scale bar = 50 µm. **(H)** Quantitative analysis of Nissl staining: upper panel shows hippocampal CA1 region, lower panel shows CA3 region. **(I)** Lactate dehydrogenase (LDH) release levels in hippocampal tissues from sh-CPEB1 and ad-CPEB1 mice and their respective controls, reflecting neuronal injury. Data are presented as mean ± SEM. *P < 0.05, **P < 0.01, ***P < 0.001, ****P < 0.0001.

To further elucidate the role of CPEB1 in epileptic seizures, we employed an adeno-associated virus (AAV)–mediated strategy to establish CPEB1 overexpression (ad-CPEB1) and knockdown (sh-CPEB1) models in the bilateral hippocampal CA1 regions of mice. The CA1 subregion was selected because of its high vulnerability to excitotoxic injury in temporal lobe epilepsy (TLE), while the CA3 region was additionally analyzed due to its strong synaptic connectivity with CA1 and its involvement in seizure propagation within the hippocampal trisynaptic circuit (38). Twenty-one days after viral injection, Western blot analysis was conducted to verify CPEB1 expression in the hippocampus and cortex. The results demonstrated a significant upregulation of CPEB1 in the ad-CPEB1 group and a marked downregulation in the sh-CPEB1 group, confirming the effectiveness of the interventions (**Supplementary Figure 4**). To evaluate the impact of CPEB1 on seizure susceptibility, pentylenetetrazol (PTZ)–induced chronic epilepsy models were established in these mice, as PTZ kindling provides a stable and quantifiable platform for assessing seizure behavior. Seizure severity was systematically evaluated using seizure scores, latency to the first generalized tonic–clonic seizure (GTC), and seizure duration. With progressive PTZ administration, mice in the sh-CPEB1 group exhibited significantly reduced seizure scores and shortened GTC duration, along with prolonged seizure latency, compared with sh-NC controls (**Figures 4A–C**), indicating decreased seizure susceptibility. In contrast, ad-CPEB1 mice displayed significantly elevated seizure scores and prolonged GTC duration relative to ad-NC controls, although latency did not show a statistically significant reduction (**Figures 4D–F**).

To assess whether CPEB1 also influences epilepsy-associated neuronal injury, histological analyses were performed using the kainic acid (KA)–induced epilepsy model, which reliably recapitulates hippocampal sclerosis and neuronal degeneration. Nissl staining revealed that CPEB1 overexpression resulted in pronounced neuronal loss and disrupted cellular architecture in both the hippocampal CA1 and CA3 regions. In contrast, sh-CPEB1 intervention markedly preserved neuronal morphology and reduced neuronal loss in these regions (**Figures 4G, H**).

Consistent with histological findings, lactate dehydrogenase (LDH) assays were conducted to quantify neuronal injury. LDH levels were significantly increased in both hippocampal and cortical tissues of ad-CPEB1 mice, whereas sh-CPEB1 mice exhibited markedly reduced LDH levels compared with their respective controls (**Figure 4I**; **Supplementary Figure S5**).

Collectively, these results demonstrate that CPEB1 exerts pro-epileptogenic and neurotoxic effects in epilepsy models. CPEB1 overexpression enhances seizure susceptibility and exacerbates neuronal death, whereas CPEB1 knockdown suppresses seizure severity and confers significant neuroprotection.

### 3.5 CPEB1 knockdown alleviates neuroinflammation and neuronal ferroptosis

**Figure 5:**
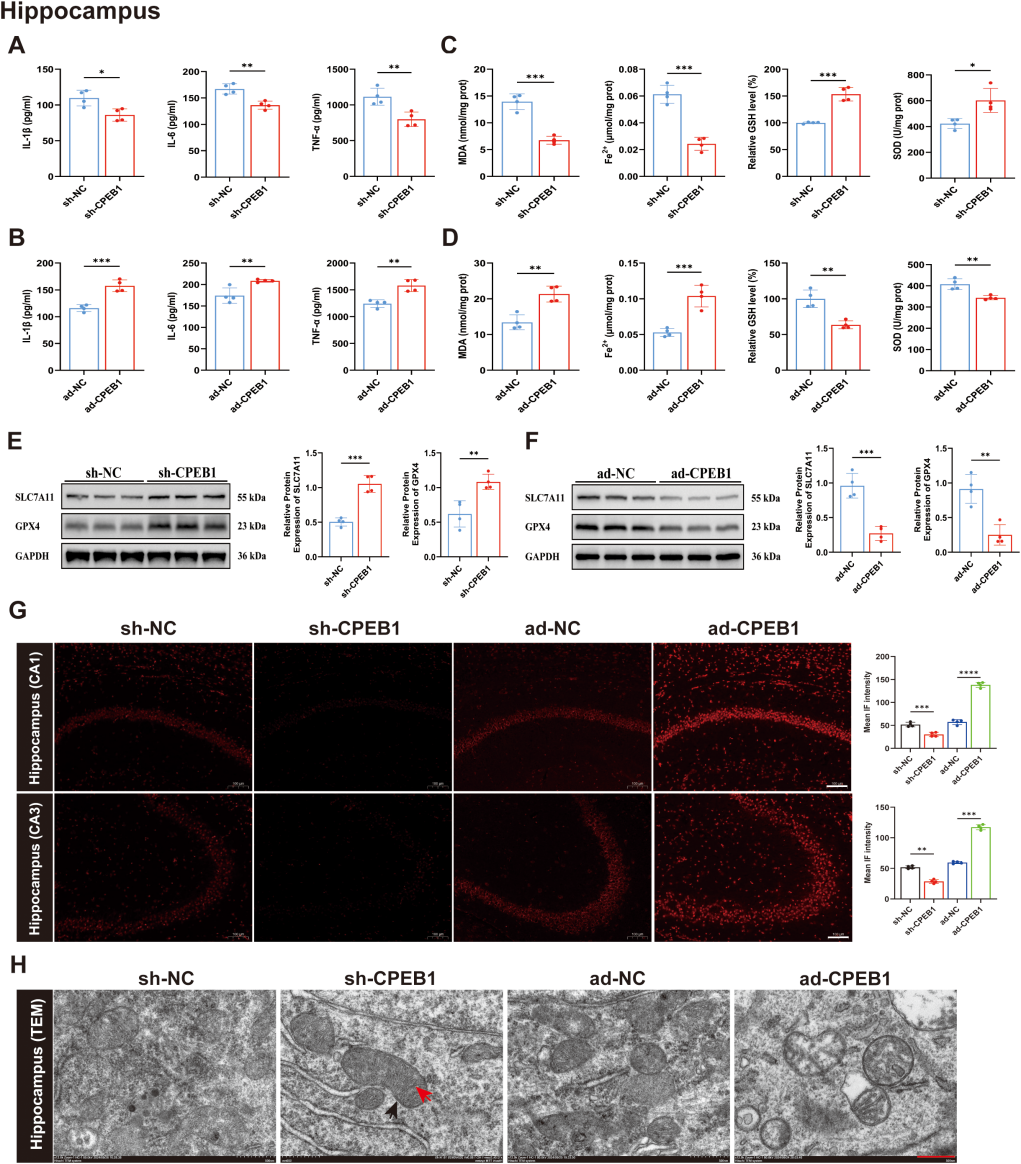
CPEB1 regulates neuroinflammation, oxidative stress, and ferroptosis in the hippocampus of KA-induced epileptic mice. **(A)** Levels of pro-inflammatory cytokines IL-1β, IL-6, and TNF-α in the hippocampus of sh-NC and sh-CPEB1 mice following kainic acid (KA)-induced epilepsy. **(B)** Levels of IL-1β, IL-6, and TNF-α in the hippocampus of ad-NC and ad-CPEB1 mice following KA-induced epilepsy. **(C)** Oxidative stress and ferroptosis-related biochemical parameters in the hippocampus of sh-NC and sh-CPEB1 mice, including malondialdehyde (MDA), Fe²^+^ content, relative glutathione **(GSH)** levels, and superoxide dismutase (SOD) activity. **(D)** Oxidative stress and ferroptosis-related biochemical parameters in the hippocampus of ad-NC and ad-CPEB1 mice. **(E)** Representative Western blot images and quantitative analysis of SLC7A11 and GPX4 protein expression in the hippocampus of sh-NC and sh-CPEB1 mice. GAPDH was used as a loading control. **(F)** Representative Western blot images and quantitative analysis of SLC7A11 and GPX4 protein expression in the hippocampus of ad-NC and ad-CPEB1 mice. **(G)** Representative fluorescence images of lipid peroxidation in hippocampal CA1 and CA3 regions, detected by DHE staining, in sh-NC, sh-CPEB1, ad-NC, and ad-CPEB1 mice. Quantification of mean fluorescence intensity is shown on the right. **(H)** Representative images of mitochondrial morphology in the hippocampus and cortex across groups. Black arrows indicate mitochondrial membranes, and red arrows indicate mitochondrial cristae. Scale bar = 500 nm. Data are presented as mean ± SEM. *P < 0.05, **P < 0.01, ***P < 0.001, ****P < 0.0001.

Elevated levels of pro-inflammatory cytokines are a hallmark of neuroinflammation, which plays a pivotal role in neuronal injury and loss following epileptic seizures (39). To assess the impact of CPEB1 on pro-inflammatory cytokine production in KA-induced epilepsy models, ELISA was performed to measure interleukin-1β (IL-1β), interleukin-6 (IL-6), and tumor necrosis factor-α (TNF-α) levels in the hippocampus and cortex. The results (**Figures 5A, B**; **Supplementary Figures S6A, B**) demonstrated that cytokine levels were significantly increased in the ad-CPEB1 group compared with the ad-NC group. In contrast, IL-1β, IL-6, and TNF-α levels were markedly reduced in the sh-CPEB1 group, with statistically significant differences relative to the sh-NC group. These findings indicate that CPEB1 exacerbates neuronal injury and loss by promoting the release of pro-inflammatory cytokines.

Furthermore, we investigated the role of CPEB1 in ferroptosis. Compared with the ad-NC group, the ad-CPEB1 group exhibited significantly elevated levels of malondialdehyde (MDA), ferrous ions (Fe²^+^), and reactive oxygen species (ROS) in both the hippocampus and cortex, accompanied by marked reductions in glutathione (GSH) content and superoxide dismutase (SOD) activity. In contrast, relative to the sh-NC group, the sh-CPEB1 group displayed decreased levels of MDA, Fe²^+^, and ROS, along with increased GSH content and SOD activity, indicating that CPEB1 knockdown mitigates oxidative stress, lipid peroxidation, and iron accumulation (**Figures 5C, D, G**; **Supplementary Figures S6C, D, G**).

We next investigated the expression of the key ferroptosis inhibitors SLC7A11 and GPX4. Western blot analysis revealed that SLC7A11 and GPX4 levels were significantly upregulated in the hippocampus and cortex of the sh-CPEB1 group, whereas they were markedly downregulated in the ad-CPEB1 group, with statistically significant differences compared to the respective controls (**Figures 5E, F**; **Supplementary Figures S6E, F**). These findings indicate that CPEB1 may facilitate ferroptosis by negatively regulating the SLC7A11/GPX4 pathway. Consistent with these results, transmission electron microscopy (TEM) revealed distinct ultrastructural changes in hippocampal and cortical neuronal mitochondria across groups. In the ad-CPEB1 group, neurons exhibited classical ferroptotic features, including mitochondrial shrinkage, reduced cristae, and increased membrane density. In contrast, neurons in the sh-CPEB1 group displayed relatively preserved mitochondrial morphology with markedly attenuated pathological alterations (**Figure 5H**; **Supplementary Figure S6H**).

In summary, CPEB1 exacerbates neuronal injury in epilepsy models by amplifying neuroinflammation and induces mitochondrial dysfunction and neuronal ferroptosis through suppression of the SLC7A11/GPX4 pathway. Conversely, CPEB1 knockdown confers robust neuroprotective effects. Collectively, these findings establish CPEB1 as a critical regulator of both neuroinflammation and ferroptosis.

### 3.6 CPEB1 promotes NRF2 degradation, thereby promoting neuroinflammation and suppressing downstream anti-ferroptotic factors

**Figure 6:**
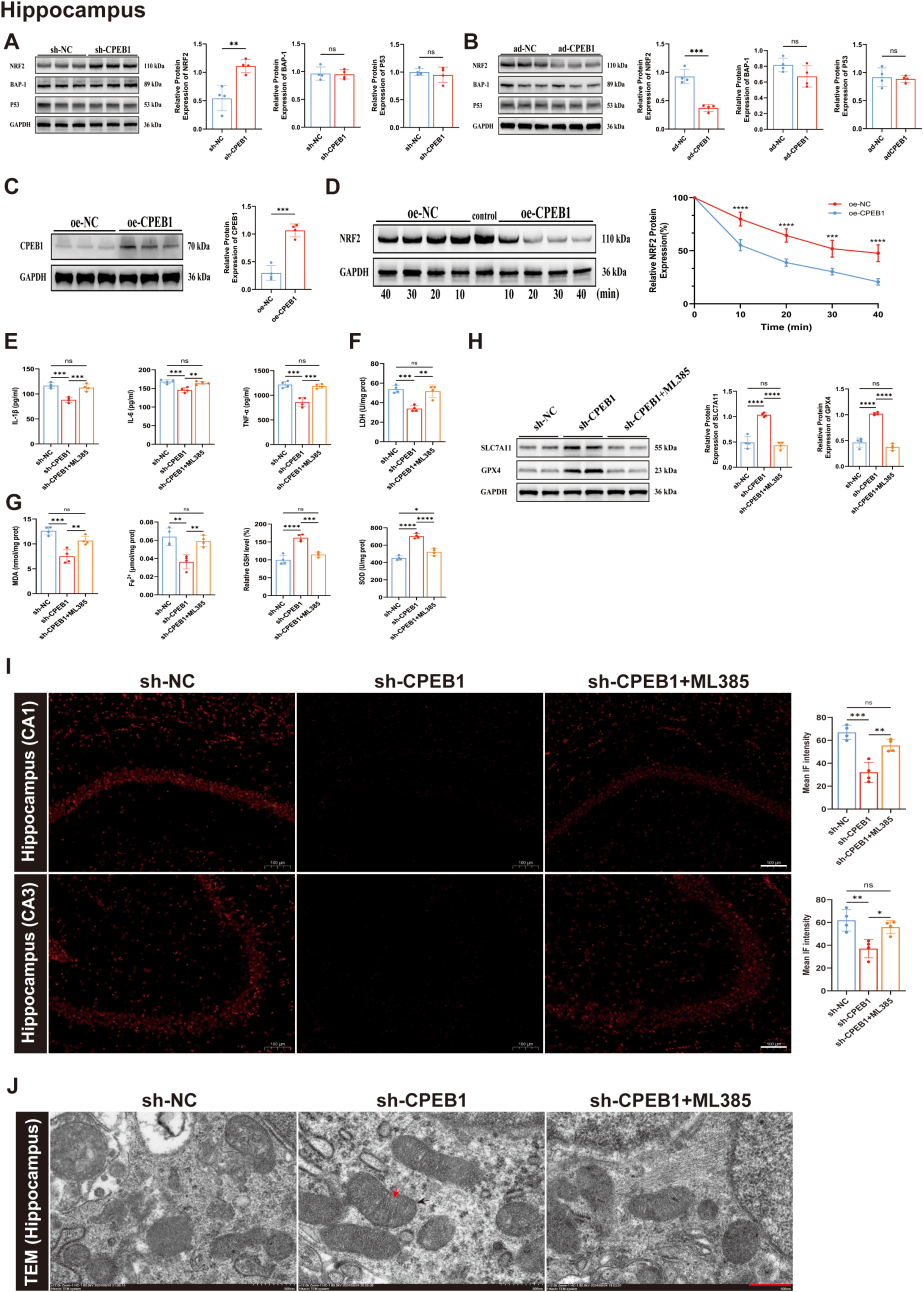
CPEB1 regulates ferroptosis through the NRF2 pathway in the hippocampus of KA-induced epileptic mice. **(A)** Representative Western blot images and quantitative analysis of NRF2, BAP1, and P53 protein expression in the hippocampus of sh-NC and sh-CPEB1 mice following KA-induced epilepsy. **(B)** Representative Western blot images and quantitative analysis of NRF2, BAP1, and P53 protein expression in the hippocampus of ad-NC and ad-CPEB1 mice following KA-induced epilepsy. **(C)** Validation of CPEB1 overexpression efficiency by Western blot analysis in hippocampal tissues. **(D)** Cycloheximide (CHX) chase assay showing NRF2 protein stability in oe-NC and oe-CPEB1 groups. Quantification of relative NRF2 protein levels at the indicated time points is shown on the right. **(E)** Levels of pro-inflammatory cytokines IL-1β, IL-6, and TNF-α in the hippocampus of sh-NC, sh-CPEB1, and sh-CPEB1+ML385 mice following KA-induced epilepsy. **(F)** LDH release levels in the hippocampus of sh-NC, sh-CPEB1, and sh-CPEB1+ML385 mice. **(G)** Oxidative stress and ferroptosis-related biochemical indices in the hippocampus, including malondialdehyde (MDA), Fe²^+^ content, relative glutathione (GSH) levels, and superoxide dismutase (SOD) activity. **(H)** Representative Western blot images and quantitative analysis of SLC7A11 and GPX4 protein expression in the hippocampus of sh-NC, sh-CPEB1, and sh-CPEB1 + ML385 mice. **(I)** Representative dihydroethidium (DHE) staining images showing reactive oxygen species (ROS) accumulation in the hippocampal CA1 and CA3 regions of sh-NC, sh-CPEB1, and sh-CPEB1+ML385 mice. Quantification of mean fluorescence intensity is shown on the right. **(J)** Representative images of mitochondrial morphology in the hippocampus and cortex across groups. Black arrows indicate mitochondrial membranes, and red arrows indicate mitochondrial cristae. Scale bar = 500 nm. Data are presented as mean ± SEM. ns, not significant; *P < 0.05, **P < 0.01, ***P < 0.001, ****P < 0.0001.

Numerous studies have established that SLC7A11 and GPX4 play essential roles in the ferroptosis pathway. SLC7A11, a cystine/glutamate antiporter, transports extracellular cystine into cells, thereby supplying substrates for glutathione (GSH) synthesis. GPX4, a GSH-dependent glutathione peroxidase, reduces lipid peroxides and prevents their accumulation. The synergistic activity of these two molecules markedly decreases intracellular lipid peroxidation, thereby effectively suppressing ferroptosis (40, 41). Previous studies have also shown that SLC7A11 expression is regulated by multiple upstream factors, including p53, NRF2, and BAP-1 (42–44). To identify potential downstream factors through which CPEB1 might regulate SLC7A11, we first performed Western blot analysis. In the hippocampus and cortex of KA model mice, both ad-CPEB1 and sh-CPEB1 interventions significantly altered NRF2 protein levels, whereas no significant changes were observed in p53 or BAP-1 (**Figures 6A, B**; **Supplementary Figures S7A, B**). We next used an *in vitro* model to determine whether CPEB1 promotes NRF2 degradation. In HT22 cells, CPEB1 was overexpressed and exposed to 5 mM glutamate (Glu) to mimic epilepsy-related excitotoxicity. Transfection efficiency was confirmed (**Figure 6C**). A cycloheximide (CHX) chase assay demonstrated that CPEB1 overexpression markedly accelerated NRF2 degradation, reducing its half-life from approximately 30 minutes in the control group to 10 minutes (**Figure 6D**). To clarify whether CPEB1 regulates the NRF2 pathway at the transcriptional or post-translational level, we examined the mRNA expression of NFE2L2 (NRF2) and its downstream antioxidant targets SLC7A11 and GPX4 in hippocampal and cortical tissues from sh-NC, sh-CPEB1, ad-NC, and ad-CPEB1 mice. Despite pronounced changes in NRF2 protein abundance detected by Western blot, qRT-PCR analysis revealed no significant differences in NFE2L2 mRNA expression among groups (**Supplementary Figure 8**), indicating that CPEB1 does not impact NRF2 transcription. Combined with the CHX chase results showing accelerated NRF2 degradation following CPEB1 overexpression, these findings support a model in which CPEB1 primarily regulates NRF2 stability at the post-translational level. In contrast, SLC7A11 and GPX4 transcript levels displayed a pattern consistent with NRF2 functional activity. CPEB1 knockdown significantly elevated SLC7A11 and GPX4 mRNA levels, whereas CPEB1 overexpression resulted in their transcriptional suppression (**Supplementary Figure 8**). These data demonstrate that loss of NRF2 protein triggered by CPEB1 effectively reduces its transcriptional activity, ultimately weakening the SLC7A11/GPX4-dependent antioxidant defense system.

Furthermore, to investigate the role of NRF2, we administered the NRF2-specific inhibitor ML385 in KA model mice with sh-CPEB1 intervention. ELISA analysis revealed that, compared with the sh-CPEB1 group, the sh-CPEB1+ML385 group exhibited significantly elevated levels of IL-1β, IL-6, TNF-α, and LDH in both the hippocampus and cortex (**Figures 6E, F**; **Supplementary Figures S7C, D**). With respect to ferroptosis-related markers, the sh-CPEB1+ML385 group showed increased MDA and Fe²^+^ levels, accompanied by reduced GSH content and SOD activity (**Figure 6G**; **Supplementary Figure S7E**). Western blot analysis further demonstrated that ML385 reversed the upregulation of SLC7A11 and GPX4 induced by sh-CPEB1 in the hippocampus and cortex (**Figure 6H**; **Supplementary Figure S7F**). Consistently, DHE fluorescence staining revealed that ML385 treatment markedly increased ROS levels in the hippocampal CA1 and CA3 regions, as well as in the cortex (**Figure 6I**, **S7G**). Transmission electron microscopy provided additional confirmation, showing that ML385 treatment aggravated mitochondrial shrinkage and structural damage in hippocampal and cortical neurons (**Figure 6J**; **Supplementary Figure S7H**). In summary, both *in vivo* and *in vitro* experiments consistently demonstrated that CPEB1 promotes NRF2 degradation, enhances the release of pro-inflammatory cytokines, and suppresses the downstream SLC7A11/GPX4 pathway, thereby exacerbating neuronal ferroptosis in TLE.

### 3.7 CPEB1 aggravates neuroinflammation and neuronal ferroptosis by negatively regulating the SIRT1/NRF2 signaling axis

**Figure 7:**
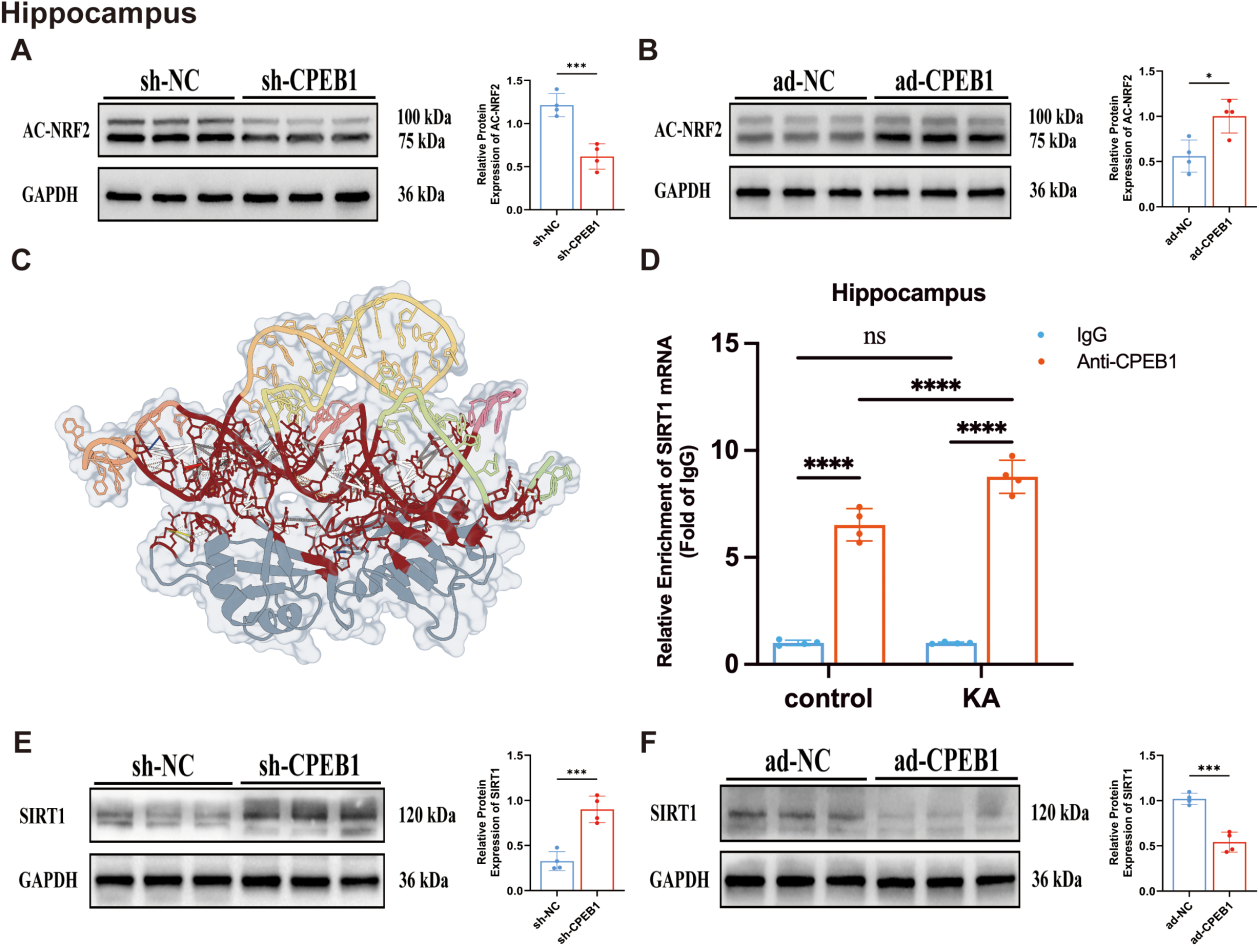
CPEB1 regulates NRF2 acetylation and SIRT1 expression in the hippocampus of KA-induced epileptic mice. **(A)** Representative Western blot images and quantitative analysis showing acetylated NRF2 (Ac-NRF2) protein levels in the hippocampus of sh-NC and sh-CPEB1 mice following kainic acid (KA)-induced epilepsy. **(B)** Representative Western blot images and quantitative analysis showing Ac-NRF2 protein levels in the hippocampus of ad-NC and ad-CPEB1 mice following KA-induced epilepsy. **(C)** Molecular docking model (HDOCK) visualizing the high-affinity interaction interface between the CPEB1 RRM domain and the SIRT1 mRNA 3’UTR. **(D)** RNA immunoprecipitation (RIP) assay showing enrichment of SIRT1 mRNA pulled down by anti-CPEB1 antibody or IgG control in the hippocampus of control and KA-treated mice. **(E)** Representative Western blot images and quantitative analysis of SIRT1 protein expression in the hippocampus of sh-NC and sh-CPEB1 mice. **(F)** Representative Western blot images and quantitative analysis of SIRT1 protein expression in the hippocampus of ad-NC and ad-CPEB1 mice. Data are presented as mean ± SEM. ns, not significant; *P < 0.05, ***P < 0.001, ****P < 0.0001.

Previous studies have demonstrated that the stability of NRF2 protein is regulated by its acetylation modification (45). To determine whether CPEB1 influences NRF2 degradation through acetylation, we examined the effects of CPEB1 overexpression and knockdown on NRF2 acetylation at the Lys599 site in the hippocampus and cortex of KA model mice. Western blot analysis revealed that sh-CPEB1 significantly reduced NRF2 Lys599 acetylation levels in both the hippocampus and cortex (**Figure 7A**; **Supplementary Figure S9B**), whereas ad-CPEB1 markedly enhanced acetylation at this site (**Figure 7B**; **Supplementary Figure S9C**). These findings suggest that CPEB1 promotes NRF2 acetylation, thereby reducing its stability and contributing to the regulation of neuroinflammation and ferroptosis. We next sought to identify potential upstream regulators of NRF2 acetylation. Bioinformatics analysis (**Figure 2D**) identified SIRT1 as a candidate molecule strongly associated with neuronal ferroptosis in TLE. Prior studies have shown that SIRT1 activation can deacetylate NRF2, enhancing its stability and activity (45, 46).

Molecular docking using HDOCK revealed a strong structural complementarity between the RRM domain of CPEB1 and the 3’ UTR of SIRT1 mRNA (**Figure 7C**). The top-ranked docking model (Rank 1) demonstrated exceptional binding stability (score: −319.36; confidence: 0.9673), supporting a biologically plausible direct interaction (**Table 5**). The RNA underwent significant conformational adjustment (RMSD: 69.30 Å), indicating an induced-fit mode of binding. Structural analysis revealed 113 atomic contacts forming a multi-layered recognition network (**Table 6**). This includes (1) a dual electrostatic anchoring mechanism via Lys279 and Arg395 that clamp the RNA’s 5’ and 3’ ends (2); a central aromatic recognition core involving π - π stacking between Tyr389 and U16, and Phe353 and U74; and (3) an extensive hydrogen bond network comprising 29 strong H-bonds, such as Lys388–U16 and Arg275–G49, that stabilize the U-rich motif recognition. To validate the molecular docking results and test whether CPEB1 physically binds to SIRT1 mRNA, we performed RNA immunoprecipitation (RIP) assays using hippocampal and cortical tissues. As shown in **Figure 7D**; **Supplementary Figures 9A**, -anti-CPEB1 antibody significantly enriched SIRT1 mRNA compared to the IgG negative control in both the hippocampus (**Figure 7D**) and cortex (**Supplementary Figure 9A**). Notably, the enrichment level of SIRT1 mRNA bound to CPEB1 was markedly higher in the KA-induced epilepsy group (~8–11 fold) than in the control group (~6 fold) (p < 0.0001), consistent with the upregulated expression of CPEB1 in epileptic tissues. These findings directly demonstrate that CPEB1 physically interacts with SIRT1 mRNA, supporting its role as a post-transcriptional regulatory factor. Subsequently, to test whether CPEB1 regulates SIRT1 protein expression, we performed Western blot analysis. The results demonstrated that sh-CPEB1 significantly upregulated SIRT1 levels in the hippocampus and cortex of epileptic mice (**Figure 7E**; **Supplementary Figure S9D**), whereas ad-CPEB1 led to reduced SIRT1 expression (**Figure 7F**; **Supplementary Figure S9E**).

**Table 5:** Summary of the top 10 docking models for the CPEB1-SIRT1 complex predicted by HDOCK.

Rank	Docking score	Confidence score	Ligand RMSD (Å)	Interface residues
1	-319.36	0.9673	69.30	Selected Model
2	-316.48	0.9654	65.86	Model_2
3	-284.94	0.9370	68.87	Model_3
4	-282.39	0.9339	56.62	Model_4
5	-281.74	0.9331	72.85	Model_5
6	-279.34	0.9300	59.39	Model_6
7	-275.36	0.9246	63.99	Model_7
8	-274.67	0.9237	74.34	Model_8
9	-272.80	0.9210	61.01	Model_9
10	-271.07	0.9184	65.66	Model_10

**Table 6:** Comprehensive list of key residue interactions at the CPEB1-SIRT1 interface.

Protein residue	RNA nucleotide	Interaction type	Atom pair (protein - RNA)	Distance (Å)	Structural role
PRO244	C47	Strong Hydrogen Bond	O - H (O3')	2.20	Backbone Stabilization
TRP245	U48	Strong Hydrogen Bond	H (N) - OP1	2.08	Backbone Recognition
PRO267	A18	Strong Hydrogen Bond	H (N) - OP2	1.80	High-affinity Binding
HIS273	G61	π-π Stacking	CE1 - N3	4.37	Auxiliary Recognition
HIS273	U60	Strong Hydrogen Bond	NE2 - O2	3.07	Base Specificity
ARG275	G49	Strong Hydrogen Bond	H (NH2) - O4'	1.92	Ribose Fixation
ARG275	U48	Strong Hydrogen Bond	H (NH2) - O2'	2.14	Ribose Fixation
CYS276	U62	Strong Hydrogen Bond	H (N) - O4'	1.19	Strong Backbone Bond
LYS279	A46	Strong Hydrogen Bond	H (NZ) - N3	1.60	Core Anchoring
LYS279	A63	Strong Hydrogen Bond	NZ - H (N6)	2.25	Distal Binding
LYS279	C47	Ionic / H-Bond	O - H (O2')	2.50	Backbone Binding
LYS279	A63	Hydrophobic	CE - C2	3.13	Auxiliary Binding
PHE353	U74	π-π Stacking	CE2 - C2'	3.13	3'-End Recognition
PHE353	U74	Strong Hydrogen Bond	H (N) - O2'	2.41	Stacking Assistance
ASP385	A72	Strong Hydrogen Bond	OD2 - O2'	1.66	Side-chain Recognition
LYS388	U16	Strong Hydrogen Bond	O - H (N3)	1.52	CPE Core Recognition
LYS388	U71	Strong Hydrogen Bond	H (N) - O2	1.80	Loop Fixation
TYR389	U16	π-π Stacking	CZ - C5'	3.69	CPE Core Recognition
TYR389	U16	Strong Hydrogen Bond	H (N) - O3'	2.53	Stacking Assistance
PRO390	U15	Strong Hydrogen Bond	H (N) - O2'	1.20	Flanking Recognition
PRO390	U15	Hydrophobic	CA - C2'	3.27	Conformational Stabilization
ARG395	U74	Ionic Interaction	NH2 - OP1	1.31	Strong 3'-End Anchoring
ARG395	G75	Ionic Interaction	NH2 - OP2	3.53	Backbone Binding
ARG395	U74	Strong Hydrogen Bond	H (NH1) - OP1	2.03	Ionic Bond Assistance
TYR429	G75	Strong Hydrogen Bond	H (N) - OP1	2.33	Terminal Stabilization

**Figure 8:**
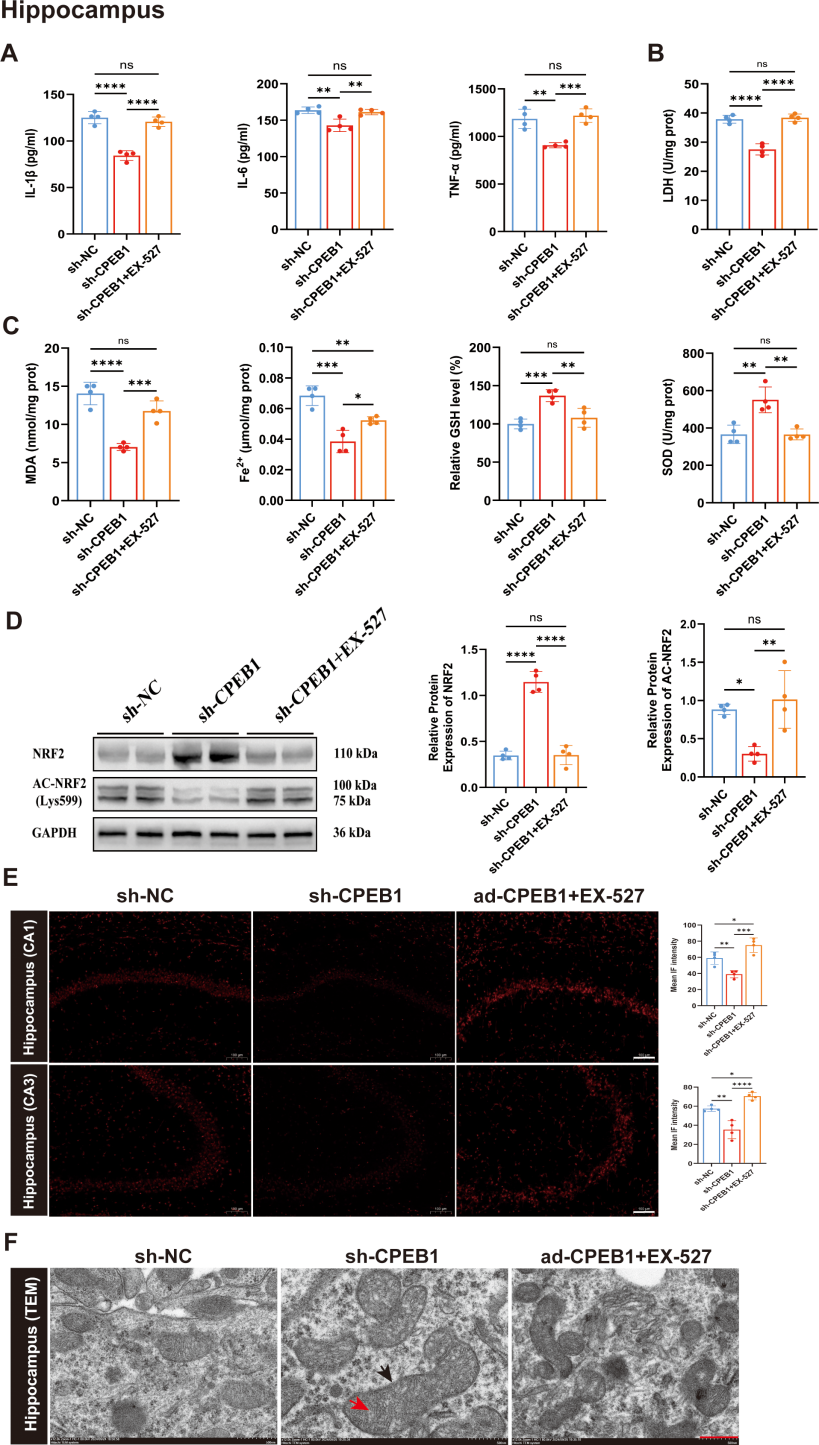
Inhibition of SIRT1 reverses the protective effects of CPEB1 knockdown on neuroinflammation and ferroptosis in the hippocampus of KA-induced epileptic mice. **(A)** Levels of pro-inflammatory cytokines IL-1β, IL-6, and TNF-α in the hippocampus of sh-NC, sh-CPEB1, and sh-CPEB1 + EX-527 mice following kainic acid (KA)-induced epilepsy. **(B)** Lactate dehydrogenase (LDH) release levels in the hippocampus of sh-NC, sh-CPEB1, and sh-CPEB1 + EX-527 mice. **(C)** Oxidative stress and ferroptosis-related biochemical indices in the hippocampus, including malondialdehyde (MDA), Fe²^+^ content, relative glutathione (GSH) levels, and superoxide dismutase (SOD) activity. **(D)** Representative Western blot images and quantitative analysis of NRF2 and acetylated NRF2 (Ac-NRF2, Lys599) protein expression in the hippocampus of sh-NC, sh-CPEB1, and sh-CPEB1 + EX-527 mice. **(E)** Representative dihydroethidium (DHE) staining images showing reactive oxygen species (ROS) accumulation in the hippocampal CA1 and CA3 regions of sh-NC, sh-CPEB1, and sh-CPEB1 + EX-527 mice. Quantification of mean fluorescence intensity is shown on the right. **(F)** Representative transmission electron microscopy (TEM) images showing mitochondrial ultrastructural alterations in hippocampal neurons. Black arrows indicate the mitochondrial outer membrane, and red arrows indicate mitochondrial cristae. Disruption of mitochondrial membranes and loss of cristae integrity are characteristic features of ferroptosis. Data are presented as mean ± SEM. ns, not significant; *P < 0.05, **P < 0.01, ***P < 0.001, ****P < 0.0001.

Next, to investigate the role of SIRT1, the SIRT1-specific inhibitor EX-527 was administered to sh-CPEB1–treated mice, and levels of IL-1β, IL-6, TNF-α, and LDH in the hippocampus and cortex were assessed. The results demonstrated that EX-527 treatment significantly increased IL-1β, IL-6, TNF-α, and LDH levels (**Figures 8A, B**; **Supplementary Figures S10A, B**). In addition, compared with the sh-CPEB1 group, the sh-CPEB1+EX-527 group exhibited markedly elevated MDA and Fe²^+^ levels, accompanied by significant reductions in GSH content and SOD activity (**Figure 8C**; **Supplementary Figure S10C**).

In addition, we assessed total NRF2 protein levels and Lys599 acetylation in the hippocampus and cortex. The results demonstrated that sh-CPEB1 significantly increased NRF2 protein levels while reducing its acetylation. However, treatment with EX-527 completely reversed these effects: NRF2 levels decreased, and Lys599 acetylation was restored to control levels (**Figure 8D**; **Supplementary Figure S10D**). DHE fluorescence staining further revealed that EX-527 treatment markedly elevated ROS levels in the hippocampal CA1 and CA3 regions, as well as in the cortex (**Figure 8E**; **Supplementary Figure S10E**). Transmission electron microscopy confirmed that EX-527 aggravated mitochondrial shrinkage and structural damage in hippocampal and cortical neurons (**Figure 8F**; **Supplementary Figure S10F**).

In summary, this study demonstrates that CPEB1 binds to the 3’ UTR of SIRT1 mRNA to repress its translation, leading to reduced SIRT1 protein expression. This, in turn, enhances NRF2 acetylation and accelerates its degradation, thereby activating neuroinflammatory responses, inhibiting the SLC7A11/GPX4 anti-ferroptotic pathway, and ultimately exacerbating neuronal ferroptosis in TLE.

## 4 Discussion

The present study systematically demonstrates that cytoplasmic polyadenylation element-binding protein 1 (CPEB1) functions as a pivotal regulator of neuroinflammation and ferroptosis in temporal lobe epilepsy (TLE). Through the integration of multi-omics analyses with *in vivo* and *in vitro* experimental validation, we found that CPEB1 overexpression exacerbates seizure susceptibility, neuronal injury, and oxidative stress, whereas its knockdown confers robust neuroprotection. Mechanistically, CPEB1 suppresses SIRT1 expression and enhances NRF2 acetylation, thereby accelerating NRF2 degradation and impairing the SLC7A11/GPX4 antioxidant defense system. Collectively, these findings reveal a previously unrecognized CPEB1/SIRT1/NRF2 signaling axis that drives ferroptosis and neuroinflammation in epilepsy, offering new conceptual and therapeutic insights into the pathogenesis of TLE.

Our findings demonstrate that CPEB1 plays a dual role in promoting both neuronal ferroptosis and neuroinflammation, two processes increasingly recognized as central determinants of epileptogenesis. The upregulation of CPEB1 observed in TLE patient samples and animal models is not merely correlative; functional manipulations confirmed its causal role in exacerbating seizure severity and neuronal loss. Mechanistically, CPEB1 negatively regulates the SIRT1/NRF2 pathway, thereby destabilizing cellular antioxidant defenses and driving excessive accumulation of ROS, iron, and lipid peroxides—hallmarks of ferroptosis. Concurrently, the suppression of SLC7A11 and GPX4 expression further heightens susceptibility to oxidative stress. In parallel, CPEB1 overexpression enhances the release of pro-inflammatory cytokines, which synergistically amplify neuronal excitability and degeneration. Together, these findings establish a mechanistic framework linking post-transcriptional regulation by CPEB1 to the cellular and molecular processes that underlie TLE. 

The present findings align with previous reports identifying ferroptosis as a critical pathogenic mechanism in neurological disorders. Recent studies have demonstrated that ferroptosis contributes to neuronal death in stroke (47), Parkinson’s disease (48), and Alzheimer’s disease (49), yet its direct role in epilepsy has remained insufficiently characterized. Limited evidence has shown increased iron deposition and ROS accumulation in epileptic hippocampi (50, 51), while treatment with ferroptosis inhibitors such as Ferrostatin-1 attenuates seizure-induced neuronal injury (10). Our study extends these observations by identifying CPEB1 as an upstream regulator of ferroptosis, thereby addressing a critical gap in the understanding of epilepsy pathogenesis.

With respect to inflammatory mechanisms, previous studies have identified IL-1β, IL-6, and TNF-α as key mediators of epileptogenesis (52, 53). In this study, we demonstrate that CPEB1 regulates cytokine release through NRF2 suppression, thereby linking redox imbalance to the amplification of inflammatory responses. This integrated perspective moves beyond earlier work that considered inflammation and oxidative stress as largely parallel processes, instead suggesting that CPEB1 orchestrates their convergence.

Notably, the identification of SIRT1 as a downstream effector of CPEB1 adds to the growing body of evidence supporting the neuroprotective functions of SIRT1. For instance, activation of SIRT1 has been shown to attenuate neuronal apoptosis and oxidative stress in epilepsy and other neurodegenerative disorders (54, 55). To our knowledge, this study is the first to demonstrate that CPEB1 negatively regulates SIRT1, thereby indirectly destabilizing NRF2. This newly uncovered pathway establishes CPEB1 as a critical upstream regulator of the SIRT1/NRF2 axis in epilepsy.

The dissection of the CPEB1-SIRT1-NRF2 axis in this study further reveals a multilayered regulatory mechanism in which post-transcriptional control drives downstream protein stability and signaling disruption. A key mechanistic insight arising from our findings is that CPEB1, traditionally characterized as a cytoplasmic polyadenylation regulator, facilitates NRF2 protein loss without altering its transcript abundance. Our combined multi-omics profiling and molecular evidence support a sequential regulatory model in which CPEB1 suppresses SIRT1 translation, resulting in reduced deacetylation capacity and destabilization of NRF2. The consequent hyperacetylation accelerates NRF2 ubiquitin–proteasomal degradation, thereby impairing its transcriptional activity. This relay-like hierarchy—where translational repression of SIRT1 governs NRF2 protein fate—represents a novel regulatory paradigm distinct from mechanisms reported in cancer biology, where CPEB1 modulates NRF2 via the p62/SQSTM1–Keap1 pathway (21). Moreover, the downstream reduction of SLC7A11 and GPX4 transcription demonstrates that this upstream post-transcriptional interference propagates to the genomic output level, ultimately compromising neuronal antioxidant defense against seizure-induced oxidative stress. These findings position CPEB1 not merely as a translational regulator, but as a master switch coupling RNA regulation to metabolic deterioration in TLE.

From a translational perspective, our findings suggest that targeting the CPEB1-SIRT1-NRF2 axis offers a promising multi-modal therapeutic strategy. Firstly, direct inhibition of CPEB1 could be achieved through nucleic acid-based therapies. Antisense oligonucleotides (ASOs) targeting CPEB1 mRNA represent a viable approach, given the clinical success of ASOs in other CNS disorders (e.g., Nusinersen) (56, 57). Alternatively, viral vector-mediated delivery of shRNA or emerging CRISPR interference (CRISPRi) technologies could provide long-term suppression of neuronal CPEB1 activity (58, 59). Secondly, for pharmacological intervention, repurposing existing drugs that modulate downstream nodes of this pathway is clinically feasible. Our data implies that NRF2 activators, such as Dimethyl fumarate (DMF) (60), which is FDA-approved for multiple sclerosis, could bypass CPEB1-mediated suppression to restore antioxidant defenses. Similarly, SIRT1 agonists like Resveratrol (61) or SRT1720 (54) may counteract the translational repression imposed by CPEB1. Furthermore, given the central role of iron accumulation in ferroptosis, blood-brain barrier-permeable iron chelators such as Deferiprone (62) could be used as an adjunct therapy to directly sequester labile iron and block lipid peroxidation. By linking these pharmacological agents to the CPEB1-driven mechanism, our study provides a theoretical framework for their application in drug-resistant epilepsy.

Several limitations should be acknowledged. First, although patient tissues and two complementary animal models were analyzed, the limited number of human specimens may restrict the generalizability of our findings. Second, while this study delineated the CPEB1/SIRT1/NRF2 axis, other downstream targets of CPEB1 cannot be excluded, and unbiased approaches such as ribosome profiling may be required to comprehensively characterize its translational regulatory network. Third, although the pharmacological inhibitors of NRF2 and SIRT1 used in this study are widely applied, potential off-target effects cannot be ruled out; genetic manipulations would provide more definitive validation. Finally, given the heterogeneity of epilepsy, it remains unclear whether CPEB1 regulation is a universal mechanism across different subtypes or is specific to TLE.

Future research should focus on expanding clinical validation in larger, well-characterized patient cohorts, including longitudinal studies to track CPEB1 expression dynamics across disease progression. The development and evaluation of small molecules or antisense oligonucleotides targeting CPEB1 may hold translational potential. In addition, the integration of spatial transcriptomics with multi-omics approaches will enable a deeper dissection of cellular heterogeneity and microenvironmental interactions underlying CPEB1-driven pathology.

In conclusion, this study identifies CPEB1 as a critical driver of neuroinflammation and ferroptosis in TLE through suppression of the SIRT1/NRF2 pathway. By establishing a mechanistic link between post-transcriptional regulation, oxidative stress, and inflammatory amplification, our findings expand the conceptual framework of epileptogenesis and highlight new therapeutic opportunities. Future research should prioritize validating this axis and developing pharmacological strategies to modulate it, thereby paving the way for innovative treatments for refractory epilepsy.

## 5 Conclusion

**Figure 9:**
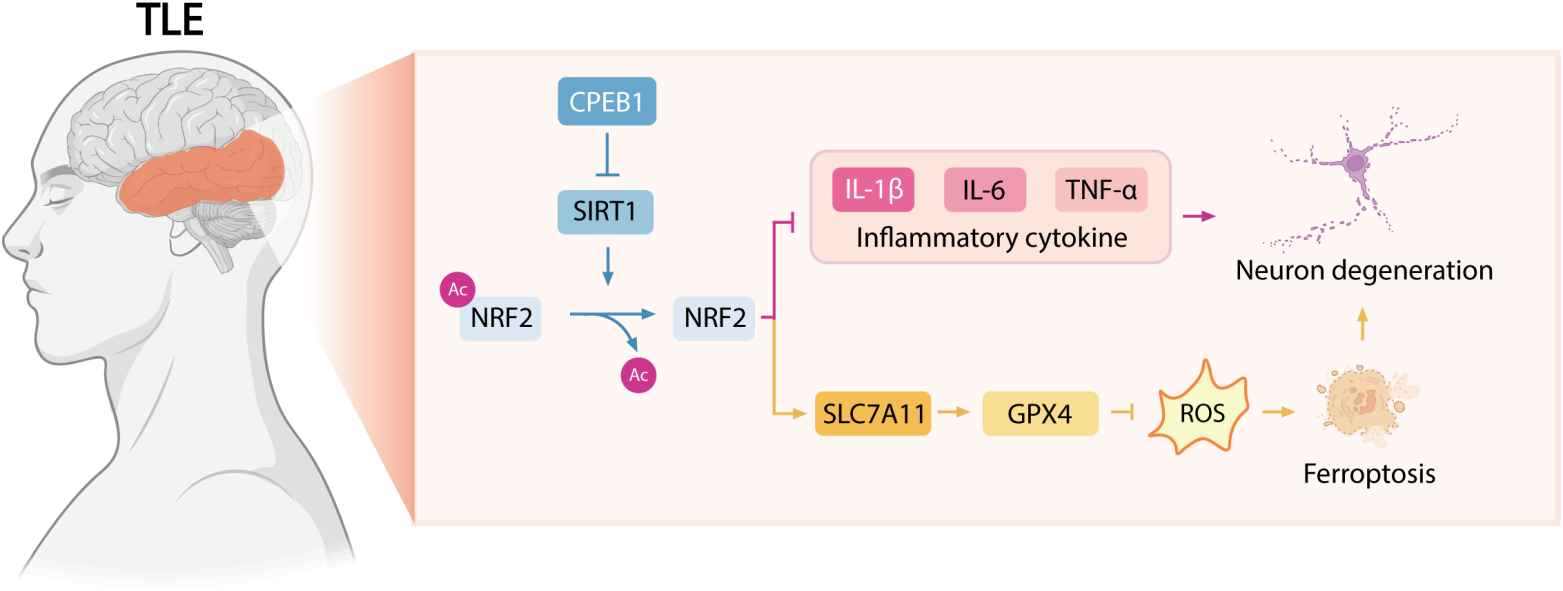
Schematic representation of the mechanism by which CPEB1 exacerbates neuronal injury in TLE through suppression of the SIRT1/NRF2 signaling pathway. CPEB1 downregulates SIRT1 expression and enhances NRF2 acetylation, thereby accelerating NRF2 degradation. Consequently, this process promotes the release of pro-inflammatory mediators while simultaneously inhibiting the SLC7A11/GPX4 axis, which facilitates neuronal ferroptosis and ultimately intensifies neuronal damage.

The findings of this study indicate that CPEB1 aggravates epileptogenesis and neuronal injury by promoting neuroinflammation and ferroptosis through the SIRT1/NRF2 signaling pathway (**Figure 9**). CPEB1 overexpression enhanced seizure susceptibility, exacerbated neuronal loss, and increased oxidative stress, whereas its knockdown exerted pronounced neuroprotective effects. Mechanistically, CPEB1 inhibited SIRT1 activity and facilitated NRF2 degradation, thereby impairing the SLC7A11/GPX4 antioxidant defense system. These results highlight the pivotal role of the CPEB1/SIRT1/NRF2 axis in epilepsy pathogenesis. Consequently, targeting this signaling pathway may represent a promising therapeutic strategy for temporal lobe epilepsy and its associated neurodegenerative processes.

## Data Availability

The scRNA-seq data used in this study are available in the Gene Expression Omnibus (GEO) repository under accession number GSE190452. The bulk RNA-seq data are deposited in the GEO repository under accession number GSE256068. All other data generated or analyzed during this study are available from the corresponding author upon reasonable request.

## Supplementary material

Supplementary Figure 1 Quality control of single-cell RNA sequencing data and dimensionality reduction analysis by PCA. **(A)** Violin plots depicting the distributions of nFeature_RNA, nCount_RNA, and percent.mt for each cell in the single-cell RNA-seq dataset. **(B)** Scatter plots of filtered nCount_RNA versus percent.mt, and of nCount_RNA versus nFeature_RNA, illustrating QC filtration thresholds and the correlation between sequencing depth and gene detection. **(C)** PCA heatmap showing the expression patterns of the top 25 genes most strongly correlated with principal components 1–4; yellow denotes upregulated expression and purple denotes downregulated expression. **(D)** Heatmap of the top 150 marker genes for each cell cluster, with yellow indicating relative upregulation and purple indicating relative downregulation.

Supplementary Figure 2 Expression and cellular localization of CPEB1 in the cortex of TLE patients and KA-induced epileptic mice. **(A)** Representative Western blot images and quantitative analysis showing CPEB1 protein expression in the cortical tissues of temporal lobe epilepsy (TLE) patients and non-epileptic control subjects. GAPDH was used as a loading control. **(B)** Representative Western blot images and quantitative analysis showing CPEB1 protein expression in the cortex of kainic acid (KA)-induced epileptic mice and control mice. **(C)** Immunofluorescence staining of CPEB1 (green), NeuN (neuronal marker, red), and DAPI (blue) in the cortical tissues of TLE patients and KA-induced epileptic mice. Line-scan analyses show fluorescence intensity profiles indicating colocalization of CPEB1 with NeuN. **(D)** Immunofluorescence staining of CPEB1 (green), GFAP (astrocyte marker, red), and DAPI (blue) in the cortical tissues of TLE patients and KA-induced epileptic mice. Line-scan analyses indicate minimal colocalization between CPEB1 and GFAP. **(E)** Immunofluorescence staining of CPEB1 (green), Iba-1 (microglial marker, red), and DAPI (blue) in the cortical tissues of TLE patients and KA-induced epileptic mice. Line-scan analyses indicate minimal colocalization between CPEB1 and Iba-1. Scale bar = 50 μm. Data are presented as mean ± SEM. ***P* < 0.01, ****P* < 0.001.

Supplementary Figure 3 Expression and cellular localization of CPEB1 in the hippocampus and cortex of PTZ-induced epileptic mice. **(A)** Representative Western blot images and quantitative analysis showing CPEB1 protein expression in the hippocampus of pentylenetetrazol (PTZ)-induced epileptic mice and control mice. GAPDH was used as a loading control. **(B)** Representative Western blot images and quantitative analysis showing CPEB1 protein expression in the cortex of PTZ-induced epileptic mice and control mice. **(C)** Immunofluorescence staining of CPEB1 (green), NeuN (neuronal marker, red), and DAPI (blue) in the cortex and hippocampal CA1 and CA3 regions of PTZ-induced epileptic mice. Line-scan analyses show fluorescence intensity profiles indicating colocalization of CPEB1 with NeuN. **(D)** Immunofluorescence staining of CPEB1 (green), GFAP (astrocyte marker, red), and DAPI (blue) in the cortex and hippocampal CA1 and CA3 regions of PTZ-induced epileptic mice. Line-scan analyses indicate minimal colocalization between CPEB1 and GFAP. **(E)** Immunofluorescence staining of CPEB1 (green), Iba-1 (microglial marker, red), and DAPI (blue) in the cortex and hippocampal CA1 and CA3 regions of PTZ-induced epileptic mice. Line-scan analyses indicate minimal colocalization between CPEB1 and Iba-1. Scale bar = 50 μm. Data are presented as mean ± SEM. ***P* < 0.01, ****P* < 0.001.

Supplementary Figure 4 Validation of CPEB1 overexpression and knockdown in the hippocampus and cortex. **(A)** Western blot detection and quantification of CPEB1 levels in the hippocampus of CPEB1 knockdown mice (n = 4). **(B)** Western blot detection and quantification of CPEB1 levels in the hippocampus of CPEB1 overexpression mice (n = 4). **(C)** Western blot detection and quantification of CPEB1 levels in the cortex of CPEB1 knockdown mice (n = 4). **(D)** Western blot detection and quantification of CPEB1 levels in the cortex of CPEB1 overexpression mice (n = 4).

Supplementary Figure 5 Effects of CPEB1 knockdown or overexpression on neuronal injury in the cortex. Lactate dehydrogenase (LDH) release levels in the cortical tissues following manipulation of CPEB1 expression. Knockdown of CPEB1 (sh-CPEB1) significantly reduced LDH release compared with sh-NC, whereas overexpression of CPEB1 (ad-CPEB1) significantly increased LDH release compared with ad-NC, indicating that CPEB1 exacerbates neuronal injury in the cortex. Data are presented as mean ± SEM. *****P* < 0.0001.

Supplementary Figure 6 CPEB1 regulates neuroinflammation, oxidative stress, and ferroptosis in the cortex of KA-induced epileptic mice. **(A)** Levels of pro-inflammatory cytokines IL-1β, IL-6, and TNF-α in the cortex of sh-NC and sh-CPEB1 mice following kainic acid (KA)-induced epilepsy. **(B)** Levels of IL-1β, TNF-α, and IL-6 in the cortex of ad-NC and ad-CPEB1 mice following KA-induced epilepsy. **(C)** Oxidative stress and ferroptosis-related biochemical indices in the cortex of sh-NC and sh-CPEB1 mice, including malondialdehyde (MDA), Fe²^+^ content, relative glutathione (GSH) levels, and superoxide dismutase (SOD) activity. **(D)** Oxidative stress and ferroptosis-related biochemical indices in the cortex of ad-NC and ad-CPEB1 mice. **(E)** Representative Western blot images and quantitative analysis of SLC7A11 and GPX4 protein expression in the cortex of sh-NC and sh-CPEB1 mice. GAPDH was used as a loading control. **(F)**Representative Western blot images and quantitative analysis of SLC7A11 and GPX4 protein expression in the cortex of ad-NC and ad-CPEB1 mice. **(G)** Representative dihydroethidium (DHE) staining images showing reactive oxygen species (ROS) accumulation in the cortex of sh-NC, sh-CPEB1, ad-NC, and ad-CPEB1 mice. Quantification of mean fluorescence intensity is shown on the right. **(H)** Representative transmission electron microscopy (TEM) images showing mitochondrial ultrastructural changes in cortical neurons. Black arrows indicate mitochondrial membranes, and red arrows indicate mitochondrial cristae, with membrane disruption and cristae loss representing characteristic features of ferroptosis. Data are presented as mean ± SEM. **P* < 0.05, ***P* < 0.01, ****P* < 0.001, *****P* < 0.0001.

Supplementary Figure 7 CPEB1 regulates NRF2 signaling and ferroptosis in the cortex of KA-induced epileptic mice. **(A)** Representative Western blot images and quantitative analysis of NRF2, BAP1, and P53 protein expression in the cortex of sh-NC and sh-CPEB1 mice following kainic acid (KA)-induced epilepsy. **(B)** Representative Western blot images and quantitative analysis of NRF2, BAP1, and P53 protein expression in the cortex of ad-NC and ad-CPEB1 mice following KA-induced epilepsy. **(C)** Levels of pro-inflammatory cytokines IL-1β, IL-6, and TNF-α in the cortex of sh-NC, sh-CPEB1, and sh-CPEB1 + ML385 mice. **(D)** Lactate dehydrogenase (LDH) release levels in the cortex of sh-NC, sh-CPEB1, and sh-CPEB1 + ML385 mice. **(E)** Oxidative stress and ferroptosis-related biochemical indices in the cortex, including malondialdehyde (MDA), Fe²^+^ content, relative glutathione (GSH) levels, and superoxide dismutase (SOD) activity. **(F)** Representative Western blot images and quantitative analysis of SLC7A11 and GPX4 protein expression in the cortex of sh-NC, sh-CPEB1, and sh-CPEB1 + ML385 mice. **(G)** Representative dihydroethidium (DHE) staining images showing reactive oxygen species (ROS) accumulation in the cortex of sh-NC, sh-CPEB1, and sh-CPEB1 + ML385 mice. Quantification of mean fluorescence intensity is shown on the right. **(H)** Representative transmission electron microscopy (TEM) images showing mitochondrial ultrastructural alterations in cortical neurons. Black arrows indicate mitochondrial membranes, and red arrows indicate mitochondrial cristae. Mitochondrial membrane disruption and cristae loss are characteristic morphological features of ferroptosis. Data are presented as mean ± SEM. ns, not significant; **P* < 0.05, ***P* < 0.01, ****P* < 0.001, *****P* < 0.0001.

Supplementary Figure 8 Quantification of NFE2L2, SLC7A11, and GPX4 mRNA expression in hippocampal and cortical tissues of CPEB1-manipulated mice. **(A, B)** Relative mRNA expression levels of NFE2L2 (NRF2), SLC7A11, and GPX4 in hippocampal tissue from sh-CPEB1 and ad-CPEB1 mice compared with their respective controls (sh-NC and ad-NC). **(C, D)** Corresponding qPCR analysis performed in cortical tissue. Gene expression values were normalized to GAPDH and analyzed using the 2^-^ΔΔCt method. Data are presented as mean ± SD (n = 4). Statistical significance was determined using one-way ANOVA with Tukey’s test. ns: not significant; *p < 0.05; **p < 0.01; ***p < 0.001; ****p < 0.0001.

Supplementary Figure 9 CPEB1 regulates SIRT1 expression and NRF2 acetylation in the cortex of KA-induced epileptic mice. (A) RNA immunoprecipitation (RIP) assay showing enrichment of SIRT1 mRNA pulled down by anti-CPEB1 antibody or IgG control in the cortex of control and kainic acid (KA)-treated mice. **(B)** Representative Western blot images and quantitative analysis of acetylated NRF2 (Ac-NRF2, Lys599) protein levels in the cortex of sh-NC and sh-CPEB1 mice. **(C)** Representative Western blot images and quantitative analysis of Ac-NRF2 protein levels in the cortex of ad-NC and ad-CPEB1 mice. **(D)** Representative Western blot images and quantitative analysis of SIRT1 protein expression in the cortex of sh-NC and sh-CPEB1 mice. **(E)** Representative Western blot images and quantitative analysis of SIRT1 protein expression in the cortex of ad-NC and ad-CPEB1 mice. Data are presented as mean ± SEM. ns, not significant; ***P* < 0.01, ****P* < 0.001, *****P* < 0.0001.

Supplementary Figure 10 Inhibition of SIRT1 reverses the effects of CPEB1 knockdown on inflammation, oxidative stress, and ferroptosis in the cortex. (A) Levels of pro-inflammatory cytokines IL-1β, IL-6, and TNF-α in the cortex of sh-NC, sh-CPEB1, and sh-CPEB1 + EX-527 mice following kainic acid (KA)–induced epilepsy. (B) Lactate dehydrogenase (LDH) release levels in the cortex of sh-NC, sh-CPEB1, and sh-CPEB1 + EX-527 mice. (C) Oxidative stress and ferroptosis-related biochemical indices in the cortex, including malondialdehyde (MDA), Fe²^+^ content, relative glutathione (GSH) levels, and superoxide dismutase (SOD) activity. (D) Representative Western blot images and quantitative analysis of NRF2 and acetylated NRF2 (Ac-NRF2, Lys599) protein expression in the cortex of sh-NC, sh-CPEB1, and sh-CPEB1 + EX-527 mice. (E) Representative dihydroethidium (DHE) staining images showing reactive oxygen species (ROS) accumulation in the cortex of sh-NC, sh-CPEB1, and sh-CPEB1 + EX-527 mice. Quantification of mean fluorescence intensity is shown on the right. (F) Representative transmission electron microscopy (TEM) images showing mitochondrial ultrastructural alterations. Black arrows indicate mitochondrial membranes, and red arrows indicate mitochondrial cristae. Disruption of mitochondrial membranes and loss of cristae are characteristic morphological features of ferroptosis. Data are presented as mean ± SEM. ns, not significant; **P* < 0.05, ***P* < 0.01, ****P* < 0.001, *****P* < 0.0001.

## References

[1] Chen Z Brodie MJ Liew D Kwan P . Treatment Outcomes in Patients With Newly Diagnosed Epilepsy Treated W ith Established and New Antiepileptic Drugs: A 30-Year Longitudinal Co hort Study. JAMA Neurol. (2018) 75:279–86. doi: 10.1001/jamaneurol.2017.3949, PMID: 29279892 PMC5885858

[2] Löscher W Potschka H Sisodiya SM Vezzani A . Drug resistance in epilepsy: clinical impact, potential mechanisms, an d new innovative treatment options. Pharmacol Rev. (2020) 72:606–38. doi: 10.1124/pr.120.019539, PMID: 32540959 PMC7300324

[3] Kai Z Zhiquan Y Zhuanyi Y Liangchao D Yu Z Shiyu F . Targeting microglial GLP1R in epilepsy: A novel approach to modulate neuroinflammation and neuronal apoptosis. Eur J Pharmacol. (2024) 981:176903. doi: 10.1016/j.ejphar.2024.176903, PMID: 39154823

[4] Hayder MA-K Majid SJ Ali IA-G Ali KA Daniel JK Mayyadah FR . Epilepsy and autophagy modulators: a therapeutic split. Autophagy. (2025) 2025:1863–87. doi: 10.1080/15548627.2025.2506292, PMID: 40375490 PMC12363515

[5] Tatiana C Leeroy B Sebastian M Alanis C Ludovic C Antoine V . Activation of lysosomal iron triggers ferroptosis in cancer. Nature. (2025) 2025:492–500. doi: 10.1038/s41586-025-08974-4, PMID: 40335696 PMC12158755

[6] Chao G Zhongying M Xingru T Kai G Wei Z Aidong W . Therapeutic time window of sodium of Danshensu on cerebral ischemia and its mechanism of inhibiting oxidative stress and ferroptosis through Nrf2 pathway. Brain Res Bull. (2025) 227:111396. doi: 10.1016/j.brainresbull.2025.111396, PMID: 40403934

[7] da Costa Caiado MJ Dolga AM den Dunnen WFA . Iron(ing) out parkinsonisms: The interplay of proteinopathy and ferroptosis in Parkinson’s disease and tau-related parkinsonisms. Redox Biol. (2025) 79:103478. doi: 10.1016/j.redox.2024.103478, PMID: 39721496 PMC11732237

[8] Thorwald MA Godoy-Lugo JA Garcia G Silva J Kim M Christensen A . Iron-associated lipid peroxidation in Alzheimer’s disease is increased in lipid rafts with decreased ferroptosis suppressors, tested by chelation in mice. Alzheimers Dement. (2025) 21:e14541. doi: 10.1002/alz.14541, PMID: 39876821 PMC11775463

[9] Mei H Wu D Yong Z Cao Y Chang Y Liang J . PM(2. 5) exposure exacerbates seizure symptoms and cognitive dysfunction by disrupting iron metabolism and the Nrf2-mediated ferroptosis pathway. Sci Total Environ. (2024) 910:168578. doi: 10.1016/j.scitotenv.2023.168578, PMID: 37981141

[10] Chen KN Guan QW Yin XX Wang ZJ Zhou HH Mao XY . Ferrostatin-1 obviates seizures and associated cognitive deficits in ferric chloride-induced posttraumatic epilepsy via suppressing ferroptosis. Free Radic Biol Med. (2022) 179:109–18. doi: 10.1016/j.freeradbiomed.2021.12.268, PMID: 34952157

[11] Soltani Khaboushan A Yazdanpanah N Rezaei N . Neuroinflammation and proinflammatory cytokines in epileptogenesis. Mol Neurobiol. (2022) 59:1724–43. doi: 10.1007/s12035-022-02725-6, PMID: 35015252

[12] Villasana-Salazar B Vezzani A . Neuroinflammation microenvironment sharpens seizure circuit. Neurobiol Dis. (2023) 178:106027. doi: 10.1016/j.nbd.2023.106027, PMID: 36736598

[13] Hashemi P Mardani P Eghbali Raz Z Saedi A Fatahi E Izapanah E . Alpha-pinene decreases the elevated levels of astrogliosis, pyroptosis, and autophagy markers in the hippocampus triggered by kainate in a R at model of temporal lobe epilepsy. Mol Neurobiol. (2025) 62:2264–76. doi: 10.1007/s12035-024-04407-x, PMID: 39096444

[14] Yan M-Q Qiu X-Y Zhang S Yu X-M Sun M-J Yang Y-Z . Neuroinflammation leads to pharmacoresistance in temporal lobe epileps y via promoting spermine degradation. Acta Pharmacol Sin. (2025) 46:2908–23. doi: 10.1038/s41401-025-01594-8, PMID: 40528032 PMC12552669

[15] Li J Wu Y Zhang D Zhang Z Li S Cheng X . The roles of cytoplasmic polyadenylation element binding protein 1 in tumorigenesis. Mini Rev Med Chem. (2024) 24:2008–18. doi: 10.2174/0113895575293544240605112838, PMID: 38879767

[16] Xiao G Chen Q Zhang X . MicroRNA-455-5p/CPEB1 pathway mediates Aβ-related learning and memory deficits in a mouse model of Alzheimer’s disease. Brain Res Bull. (2021) 177:282–94. doi: 10.1016/j.brainresbull.2021.10.008, PMID: 34678444

[17] Oe S Hayashi S Tanaka S Koike T Hirahara Y Seki-Omura R . Cytoplasmic polyadenylation element-binding protein 1 post-transcriptionally regulates fragile X mental retardation 1 expression through 3’ Untranslated region in central nervous system neurons. Front Cell Neurosci. (2022) 16:869398. doi: 10.3389/fncel.2022.869398, PMID: 35496917 PMC9051318

[18] Casañas JJ González-Corrales M Urbano-Gámez JD Alves-Sampaio A Troca-Marín JA Montesinos ML . CPEB1 is overexpressed in neurons derived from Down syndrome IPSCs and in the hippocampus of the mouse model Ts1Cje. Mol Cell Neurosci. (2019) 95:79–85. doi: 10.1016/j.mcn.2019.02.002, PMID: 30763690

[19] Wei Z Liu J Xie H Wang B Wu J Zhu Z . MiR-122-5p mitigates inflammation, reactive oxygen species and SH-SY5Y apoptosis by targeting CPEB1 after spinal cord injury via the PI3K/AKT signaling pathway. Neurochem Res. (2021) 46:992–1005. doi: 10.1007/s11064-021-03232-1, PMID: 33528808

[20] Xia YR Wei XC Li WS Yan QJ Wu XL Yao W . CPEB1, a novel risk gene in recent-onset schizophrenia, contributes to mitochondrial complex I defect caused by a defective provirus ERVWE1. World J Psychiatry. (2021) 11:1075–94. doi: 10.5498/wjp.v11.i11.1075, PMID: 34888175 PMC8613759

[21] Zhang S Huang J Lan Z Xiao Y Liao Y Basnet S . CPEB1 controls NRF2 proteostasis and ferroptosis susceptibility in pancreatic cancer. Int J Biol Sci. (2024) 20:3156–72. doi: 10.7150/ijbs.95962, PMID: 38904009 PMC11186365

[22] Xiaoying G Zhipeng Y Cong H Zhixiong L Zixiao T Jiran L . NCBP1 improves cognitive function in mice by reducing oxidative stress, neuronal loss, and glial activation after status epilepticus. Mol Neurobiol. (2023) 60:6676–88. doi: 10.1007/s12035-023-03497-3, PMID: 37474884

[23] R JR . Modification of seizure activity by electrical stimulation. II. Motor seizure. Electroencephalogr Clin Neurophysiol. (1972) 32:281–94. doi: 10.1016/0013-4694(72)90177-0, PMID: 4110397

[24] Wang S Zheng Y Jin S Fu Y Liu Y . Dioscin Protects against Cisplatin-Induced Acute Kidney Injury by Redu cing Ferroptosis and Apoptosis through Activating Nrf2/HO-1 Signaling. Antioxidants (Basel). (2022) 11:2443. doi: 10.3390/antiox11122443, PMID: 36552651 PMC9774127

[25] Singh A Venkannagari S Oh KH Zhang Y-Q Rohde JM Liu L . Small molecule inhibitor of NRF2 selectively intervenes therapeutic re sistance in KEAP1-deficient NSCLC tumors. ACS Chem Biol. (2016) 11:3214–25. doi: 10.1021/acschembio.6b00651, PMID: 27552339 PMC5367156

[26] Chen L Huang J Yao Z-M Sun X-R Tong X-H Hu M . Procyanidins Alleviated Cerebral Ischemia/Reperfusion Injury by Inhibi ting Ferroptosis via the Nrf2/HO-1 Signaling Pathway. Molecules. (2023) 28:3582. doi: 10.3390/molecules28083582, PMID: 37110816 PMC10143264

[27] Xian P Hei Y Wang R Wang T Yang J Li J . Mesenchymal stem cell-derived exosomes as a nanotherapeutic agent for amelioration of inflammation-induced astrocyte alterations in mice. Theranostics. (2019) 9:5956–75. doi: 10.7150/thno.33872, PMID: 31534531 PMC6735367

[28] Broussy S Laaroussi H Vidal M . Biochemical mechanism and biological effects of the inhibition of sile nt information regulator 1 (SIRT1) by EX-527 (SEN0014196 or selisistat). J Enzyme Inhib Med Chem. (2020) 35:1124–36. doi: 10.1080/14756366.2020.1758691, PMID: 32366137 PMC7241506

[29] Marianne RS Adeela S Tamas L Judy P Brett AB Shane AW . A potent and selective Sirtuin 1 inhibitor alleviates pathology in multiple animal and cell models of Huntington’s disease. Hum Mol Genet. (2014) 23:2995–3007. doi: 10.1093/hmg/ddu010, PMID: 24436303 PMC4031626

[30] Li Q Li Q-Q Jia J-N Sun Q-Y Zhou H-H Jin W-L . Baicalein Exerts Neuroprotective Effects in FeCl_3_-Induced P osttraumatic Epileptic Seizures via Suppressing Ferroptosis. Front Pharmacol. (2019) 10:638. doi: 10.3389/fphar.2019.00638, PMID: 31231224 PMC6568039

[31] Musheshe N Oun A Sabogal-Guáqueta AM Trombetta-Lima M Mitchel SC Adzemovic A . Pharmacological inhibition of epac1 averts ferroptosis cell death by P reserving mitochondrial integrity. Antioxidants (Basel). (2022) 11:314. doi: 10.3390/antiox11020314, PMID: 35204198 PMC8868285

[32] Liu D Zhu Y . Unveiling Smyd-2’s Role in Cytoplasmic Nrf-2 Sequestration and Ferropt osis Induction in Hippocampal Neurons After Cerebral Ischemia/Reperfus ion. Cells. (2024) 13:1969. doi: 10.3390/cells13231969, PMID: 39682718 PMC11639856

[33] Yu L Zhang C Gu L Chen H Huo Y Wang S . Hydroxysafflor yellow A and tenuigenin exhibit neuroprotection effects against focal cerebral ischemia via differential regulation of JAK2/S TAT3 and SOCS3 signaling interaction. Mol Neurobiol. (2024) 61:5584–600. doi: 10.1007/s12035-023-03896-6, PMID: 38214838

[34] Lu Y Lin M Ou S Sun L Qian K Kuang H . Astragalus polysaccharides ameliorate epileptogenesis, cognitive impairment, and neuroinflammation in a pentylenetetrazole-induced kindling mouse model. Front Pharmacol. (2024) 15:1336122. doi: 10.3389/fphar.2024.1336122, PMID: 38405667 PMC10884767

[35] Wang J Wang T Zhang Y Liu J Song J Han Y . CPEB1 enhances erastin-induced ferroptosis in gastric cancer cells by suppressing twist1 expression. IUBMB Life. (2021) 73:1180–90. doi: 10.1002/iub.2525, PMID: 34184391

[36] Roggenhofer E Muller S Santarnecchi E Melie-Garcia L Wiest R Kherif F . Remodeling of brain morphology in temporal lobe epilepsy. Brain Behav. (2020) 10:e01825. doi: 10.1002/brb3.1825, PMID: 32945137 PMC7667340

[37] Sarbisheh I Tapak L Fallahi A Fardmal J Sadeghifar M Nazemzadeh M . Cortical thickness analysis in temporal lobe epilepsy using fully Baye sian spectral method in magnetic resonance imaging. BMC Med Imaging. (2022) 22:222. doi: 10.1186/s12880-022-00949-5, PMID: 36544100 PMC9768883

[38] Ma DL Tang YC Chen PM Chia SC Jiang FL Burgunder J-M . Reorganization of CA3 area of the mouse hippocampus after pilocarpine induced temporal lobe epilepsy with special reference to the CA3-septu m pathway. J Neurosci Res. (2006) 83:318–31. doi: 10.1002/jnr.20731, PMID: 16385555

[39] Singh PK Maurya S Saadi A Shekh-Ahmad T . Targeting NOX2 mitigates seizure susceptibility, oxidative stress, and neuroinflammation in the pentylenetetrazol seizure model. Free Radic Biol Med. (2025) 235:306–16. doi: 10.1016/j.freeradbiomed.2025.05.386, PMID: 40345502

[40] Koppula P Zhuang L Gan B . Cystine transporter SLC7A11/xCT in cancer: ferroptosis, nutrient depen dency, and cancer therapy. Protein Cell. (2021) 12:599–620. doi: 10.1007/s13238-020-00789-5, PMID: 33000412 PMC8310547

[41] Seiler A Schneider M Förster H Roth S Wirth EK Culmsee C . Glutathione peroxidase 4 senses and translates oxidative stress into 1 2/15-lipoxygenase dependent- and AIF-mediated cell death. Cell Metab. (2008) 8:237–48. doi: 10.1016/j.cmet.2008.07.005, PMID: 18762024

[42] Jiang L Kon N Li T Wang S-J Su T Hibshoosh H . Ferroptosis as a p53-mediated activity during tumour suppression. Nature. (7545) 520:57–62. doi: 10.1038/nature14344, PMID: 25799988 PMC4455927

[43] Rojo de la Vega M Chapman E Zhang DD . NRF2 and the hallmarks of cancer. Cancer Cell. (2018) 34:21–43. doi: 10.1016/j.ccell.2018.03.022, PMID: 29731393 PMC6039250

[44] Affar EB Carbone M . BAP1 regulates different mechanisms of cell death. Cell Death Dis. (2018) 9:1151. doi: 10.1038/s41419-018-1206-5, PMID: 30455474 PMC6242853

[45] Ding Y-W Zhao G-J Li X-L Hong G-L Li M-F Qiu Q-M . SIRT1 exerts protective effects against paraquat-induced injury in mou se type II alveolar epithelial cells by deacetylating NRF2 *in vitro*. Int J Mol Med. (2016) 37:1049–58. doi: 10.3892/ijmm.2016.2503, PMID: 26935021

[46] Xia Y Li S Wang X Zhao B Chen S Jiang Q . Astilbin targeted Sirt1 to inhibit acetylation of Nrf2 to alleviate gr ass carp hepatocyte apoptosis caused by PCB126-induced mitochondrial k inetic and metabolism dysfunctions. Fish Shellfish Immunol. (2023) 141:109000. doi: 10.1016/j.fsi.2023.109000, PMID: 37597642

[47] Hu X Bao Y Li M Zhang W Chen C . The role of ferroptosis and its mechanism in ischemic stroke. Exp Neurol. (2024) 372:114630. doi: 10.1016/j.expneurol.2023.114630, PMID: 38056585

[48] Wang Z-L Yuan L Li W Li J-Y . Ferroptosis in Parkinson’s disease: glia-neuron crosstalk. Trends Mol Med. (2022) 28:258–69. doi: 10.1016/j.molmed.2022.02.003, PMID: 35260343

[49] Mezzanotte M Stanga S . Brain iron dyshomeostasis and ferroptosis in alzheimer’s disease patho physiology: two faces of the same coin. Aging Dis. (2024) 16:2615–40. doi: 10.14336/AD.2024.0094, PMID: 38913042 PMC12339084

[50] Chen S Zhao L Jin X Liu Q Xiao Y Xu H . Astaxanthin inhibits ferroptosis of hippocampal neurons in kainic acid-induced epileptic mice by activating the nrf2/GPX4 signaling pathway. CNS Neurosci Ther. (2025) 31:e70238. doi: 10.1111/cns.70238, PMID: 39957487 PMC11831069

[51] Li X Quan P Si Y Liu F Fan Y Ding F . The microRNA-211-5p/P2RX7/ERK/GPX4 axis regulates epilepsy-associated neuronal ferroptosis and oxidative stress. J Neuroinflamm. (2024) 21:13. doi: 10.1186/s12974-023-03009-z, PMID: 38191407 PMC10773122

[52] Aguilar-Castillo MJ Cabezudo-García P García-Martín G Lopez-Moreno Y Estivill-Torrús G Ciano-Petersen NL . A systematic review of the predictive and diagnostic uses of neuroinfl ammation biomarkers for epileptogenesis. Int J Mol Sci. (2024) 25:6488. doi: 10.3390/ijms25126488, PMID: 38928193 PMC11487433

[53] Ho Y-H Lin Y-T Wu C-WJ Chao Y-M Chang AYW Chan JYH . Peripheral inflammation increases seizure susceptibility via the induc tion of neuroinflammation and oxidative stress in the hippocampus. J BioMed Sci. (2015) 22:46. doi: 10.1186/s12929-015-0157-8, PMID: 26100815 PMC4477313

[54] Thapa R Moglad E Afzal M Gupta G Bhat AA Hassan Almalki W . The role of sirtuin 1 in ageing and neurodegenerative disease: A molec ular perspective. Ageing Res Rev. (2024) 102:102545. doi: 10.1016/j.arr.2024.102545, PMID: 39423873

[55] Zhu L Yang M Fan L Yan Q Zhang L Mu P . Interaction between resveratrol and SIRT1: role in neurodegenerative d iseases. Naunyn Schmiedebergs Arch Pharmacol. (2025) 398:89–101. doi: 10.34133/research.0912, PMID: 39105797

[56] Crooke ST Baker BF Crooke RM Liang X-H . Antisense technology: an overview and prospectus. Nat Rev Drug Discov. (2021) 20:427–53. doi: 10.1038/s41573-021-00162-z, PMID: 33762737

[57] Chen X Birey F Li M-Y Revah O Levy R Thete MV . Antisense oligonucleotide therapeutic approach for Timothy syndrome. Nature. (8009) 628:818–25. doi: 10.1038/s41586-024-07310-6, PMID: 38658687 PMC11043036

[58] McTague A Rossignoli G Ferrini A Barral S Kurian MA . Genome Editing in iPSC-Based Neural Systems: From Disease Models to Fu ture Therapeutic Strategies. Front Genome Ed. (2021) 3:630600. doi: 10.3389/fgeed.2021.630600, PMID: 34713254 PMC8525405

[59] Bendixen L Jensen TI Bak RO . CRISPR-Cas-mediated transcriptional modulation: The therapeutic promis es of CRISPRa and CRISPRi. Mol Ther. (2023) 31:1920–37. doi: 10.1016/j.ymthe.2023.03.024, PMID: 36964659 PMC10362391

[60] Okuda DT Kantarci O Lebrun-Frénay C Sormani MP Azevedo CJ Bovis F . Dimethyl fumarate delays multiple sclerosis in radiologically isolated syndrome. Ann Neurol. (2023) 93:604–14. doi: 10.1002/ana.26555, PMID: 36401339

[61] Liu X Baxley S Hebron M Turner RS Moussa C . Resveratrol attenuates CSF markers of neurodegeneration and neuroinfla mmation in individuals with alzheimer’s disease. Int J Mol Sci. (2025) 26:5044. doi: 10.3390/ijms26115044, PMID: 40507855 PMC12155158

[62] Ayton S Barton D Brew B Brodtmann A Clarnette R Desmond P . Deferiprone in alzheimer disease: A randomized clinical trial. JAMA Neurol. (2025) 82:11–8. doi: 10.1001/jamaneurol.2024.3733, PMID: 39495531 PMC11536302

