# Supplementary figures and images for: CPEB1 drives ferroptosis–neuroinflammation crosstalk in temporal lobe epilepsy via the SIRT1–NRF2 acetylation axis
Source: Front Immunol. 2026 Mar 13;17:1727784. doi: 10.3389/fimmu.2026.1727784 (PMC13021461; doi:10.3389/fimmu.2026.1727784)

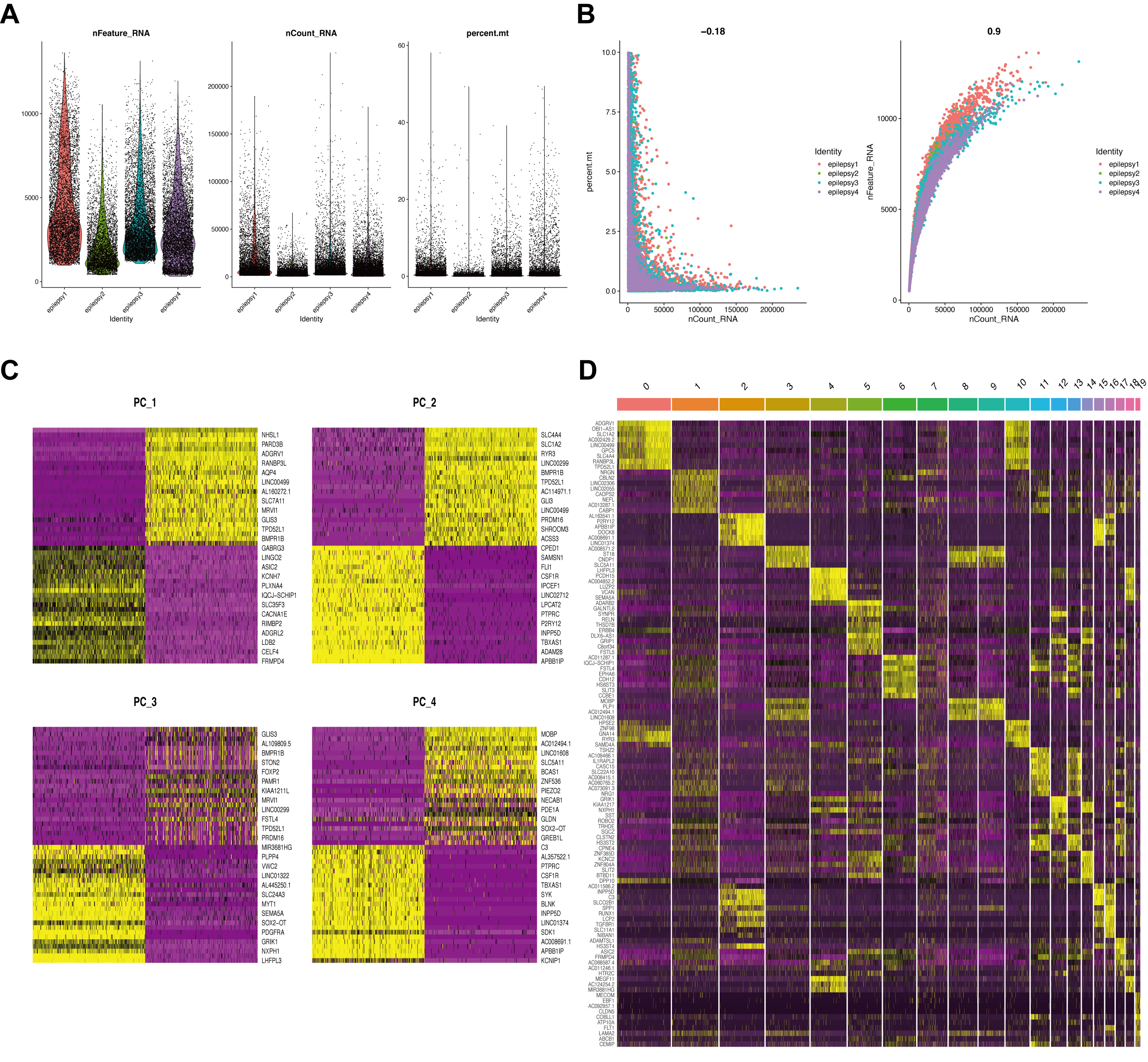

Supplement: Supplementary Figure 1 — Quality control of single-cell RNA sequencing data and dimensionality reduction analysis by PCA. (A) Violin plots depicting the distributions of nFeature_RNA, nCount_RNA, and percent.mt for each cell in the single-cell RNA-seq dataset. (B) Scatter plots of filtered nCount_RNA versus percent.mt, and of nCount_RNA versus nFeature_RNA, illustrating QC filtration thresholds and the correlation between sequencing depth and gene detection. (C) PCA heatmap showing the expression patterns of the top 25 genes most strongly correlated with principal components 1–4; yellow denotes upregulated expression and purple denotes downregulated expression. (D) Heatmap of the top 150 marker genes for each cell cluster, with yellow indicating relative upregulation and purple indicating relative downregulation. [file Image1.tif]

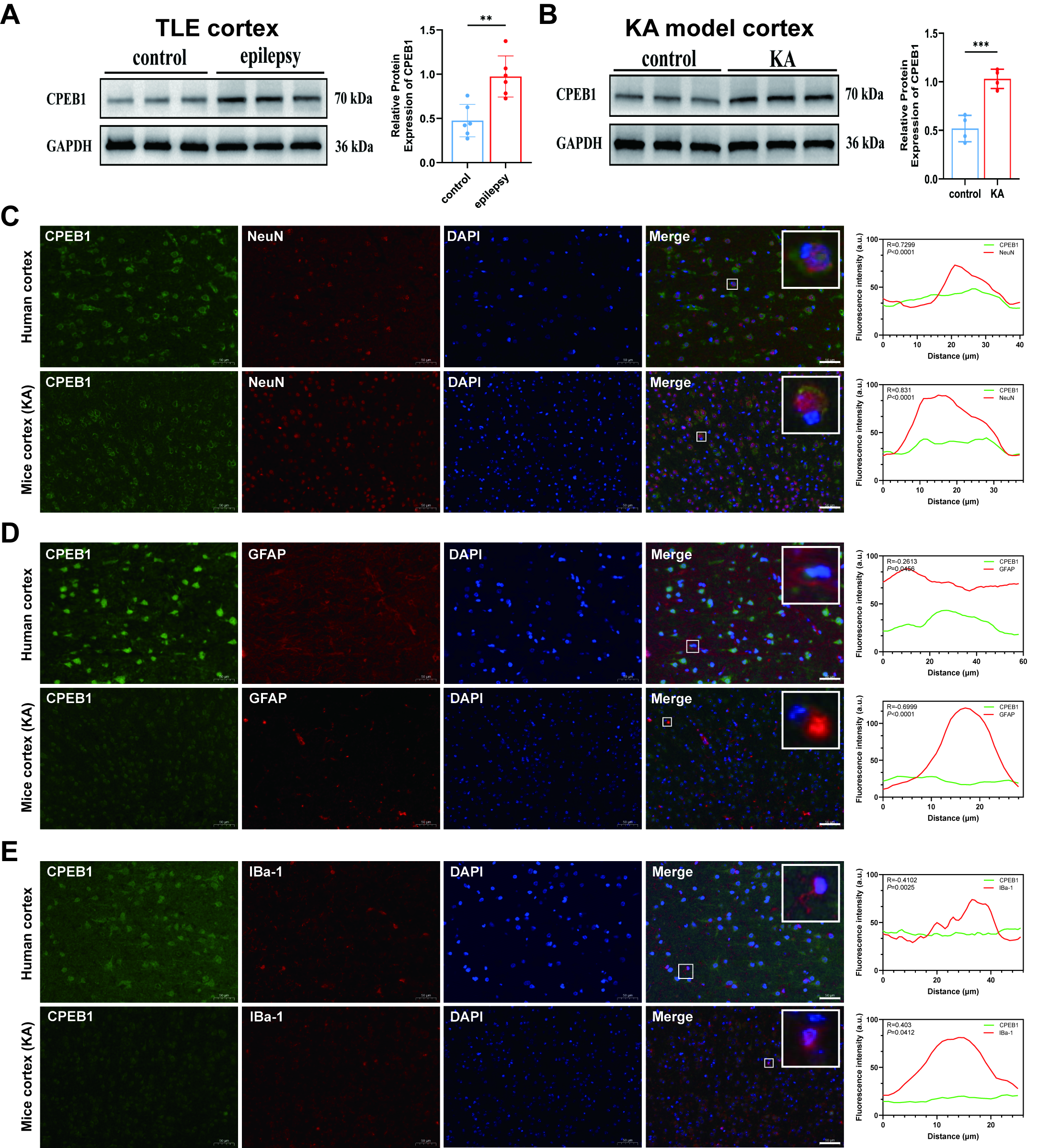

Supplement: Supplementary Figure 2 — Expression and cellular localization of CPEB1 in the cortex of TLE patients and KA-induced epileptic mice. (A) Representative Western blot images and quantitative analysis showing CPEB1 protein expression in the cortical tissues of temporal lobe epilepsy (TLE) patients and non-epileptic control subjects. GAPDH was used as a loading control. (B) Representative Western blot images and quantitative analysis showing CPEB1 protein expression in the cortex of kainic acid (KA)-induced epileptic mice and control mice. (C) Immunofluorescence staining of CPEB1 (green), NeuN (neuronal marker, red), and DAPI (blue) in the cortical tissues of TLE patients and KA-induced epileptic mice. Line-scan analyses show fluorescence intensity profiles indicating colocalization of CPEB1 with NeuN. (D) Immunofluorescence staining of CPEB1 (green), GFAP (astrocyte marker, red), and DAPI (blue) in the cortical tissues of TLE patients and KA-induced epileptic mice. Line-scan analyses indicate minimal colocalization between CPEB1 and GFAP. (E) Immunofluorescence staining of CPEB1 (green), Iba-1 (microglial marker, red), and DAPI (blue) in the cortical tissues of TLE patients and KA-induced epileptic mice. Line-scan analyses indicate minimal colocalization between CPEB1 and Iba-1. Scale bar = 50 μm. Data are presented as mean ± SEM. **P < 0.01, ***P < 0.001. [file Image2.tif]

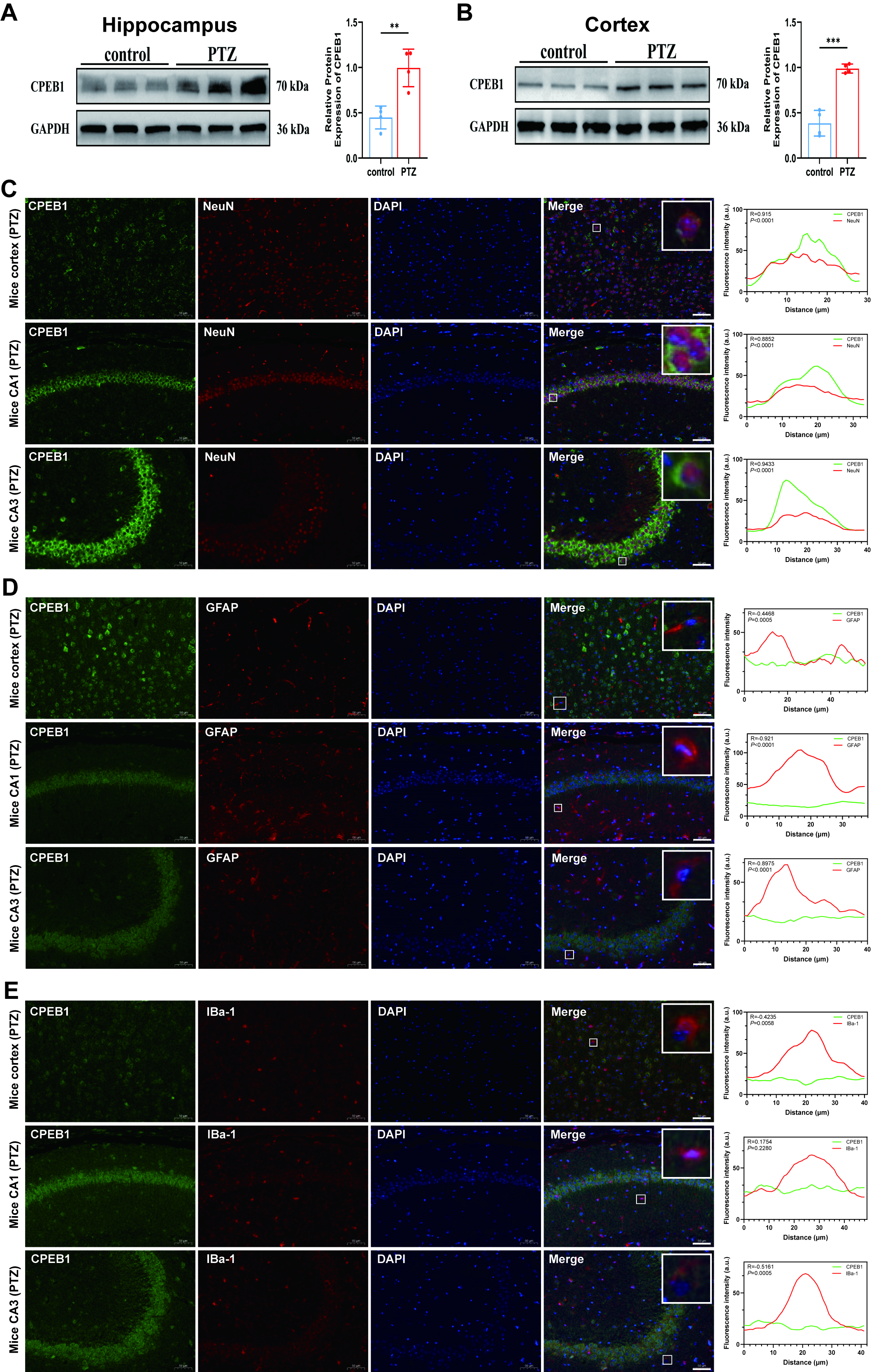

Supplement: Supplementary Figure 3 — Expression and cellular localization of CPEB1 in the hippocampus and cortex of PTZ-induced epileptic mice. (A) Representative Western blot images and quantitative analysis showing CPEB1 protein expression in the hippocampus of pentylenetetrazol (PTZ)-induced epileptic mice and control mice. GAPDH was used as a loading control. (B) Representative Western blot images and quantitative analysis showing CPEB1 protein expression in the cortex of PTZ-induced epileptic mice and control mice. (C) Immunofluorescence staining of CPEB1 (green), NeuN (neuronal marker, red), and DAPI (blue) in the cortex and hippocampal CA1 and CA3 regions of PTZ-induced epileptic mice. Line-scan analyses show fluorescence intensity profiles indicating colocalization of CPEB1 with NeuN. (D) Immunofluorescence staining of CPEB1 (green), GFAP (astrocyte marker, red), and DAPI (blue) in the cortex and hippocampal CA1 and CA3 regions of PTZ-induced epileptic mice. Line-scan analyses indicate minimal colocalization between CPEB1 and GFAP. (E) Immunofluorescence staining of CPEB1 (green), Iba-1 (microglial marker, red), and DAPI (blue) in the cortex and hippocampal CA1 and CA3 regions of PTZ-induced epileptic mice. Line-scan analyses indicate minimal colocalization between CPEB1 and Iba-1. Scale bar = 50 μm. Data are presented as mean ± SEM. **P < 0.01, ***P < 0.001. [file Image3.tif]

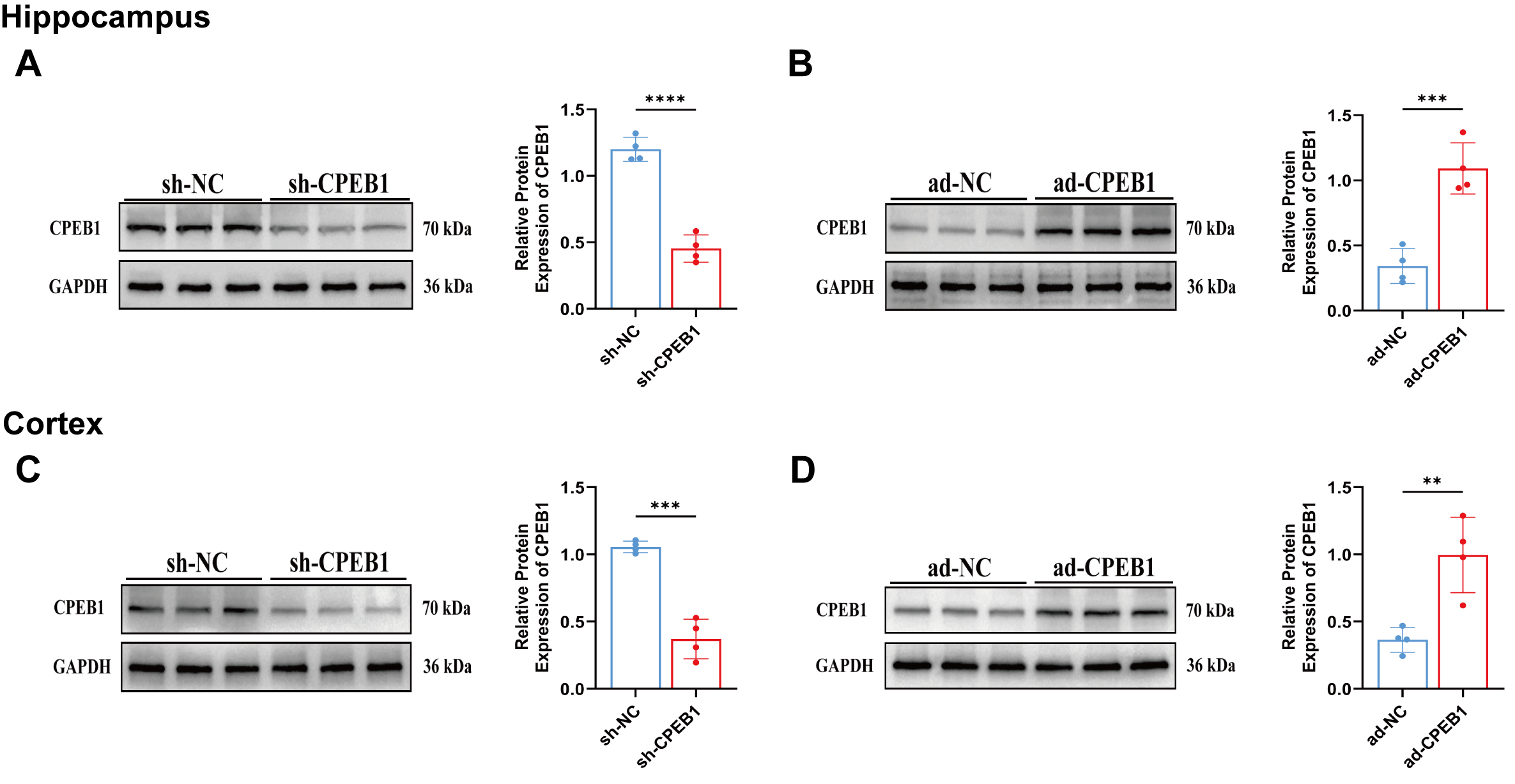

Supplement: Supplementary Figure 4 — Validation of CPEB1 overexpression and knockdown in the hippocampus and cortex. (A) Western blot detection and quantification of CPEB1 levels in the hippocampus of CPEB1 knockdown mice (n = 4). (B) Western blot detection and quantification of CPEB1 levels in the hippocampus of CPEB1 overexpression mice (n = 4). (C) Western blot detection and quantification of CPEB1 levels in the cortex of CPEB1 knockdown mice (n = 4). (D) Western blot detection and quantification of CPEB1 levels in the cortex of CPEB1 overexpression mice (n = 4). [file Image4.tif]

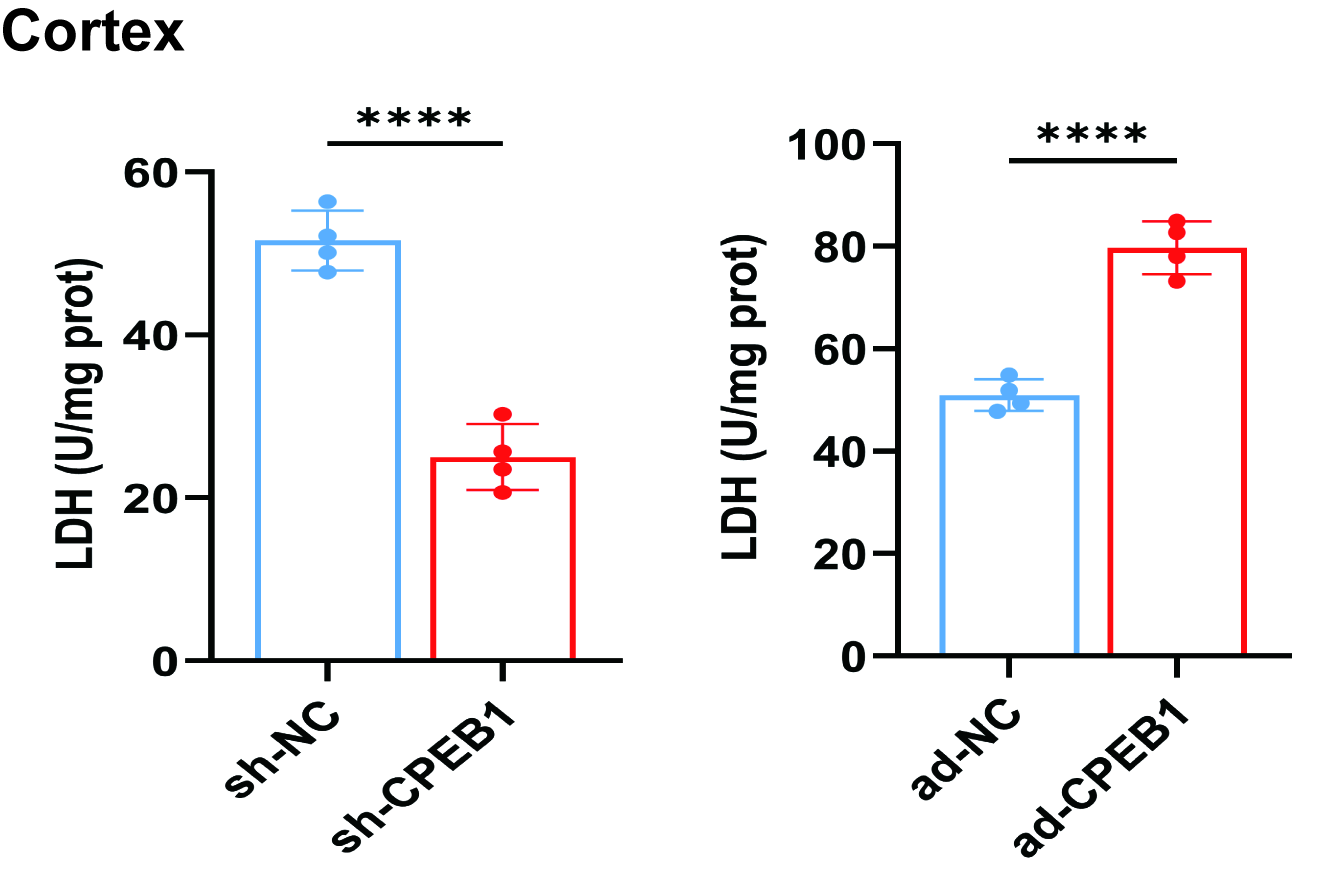

Supplement: Supplementary Figure 5 — Effects of CPEB1 knockdown or overexpression on neuronal injury in the cortex. Lactate dehydrogenase (LDH) release levels in the cortical tissues following manipulation of CPEB1 expression. Knockdown of CPEB1 (sh-CPEB1) significantly reduced LDH release compared with sh-NC, whereas overexpression of CPEB1 (ad-CPEB1) significantly increased LDH release compared with ad-NC, indicating that CPEB1 exacerbates neuronal injury in the cortex. Data are presented as mean ± SEM. ****P < 0.0001. [file Image5.tif]

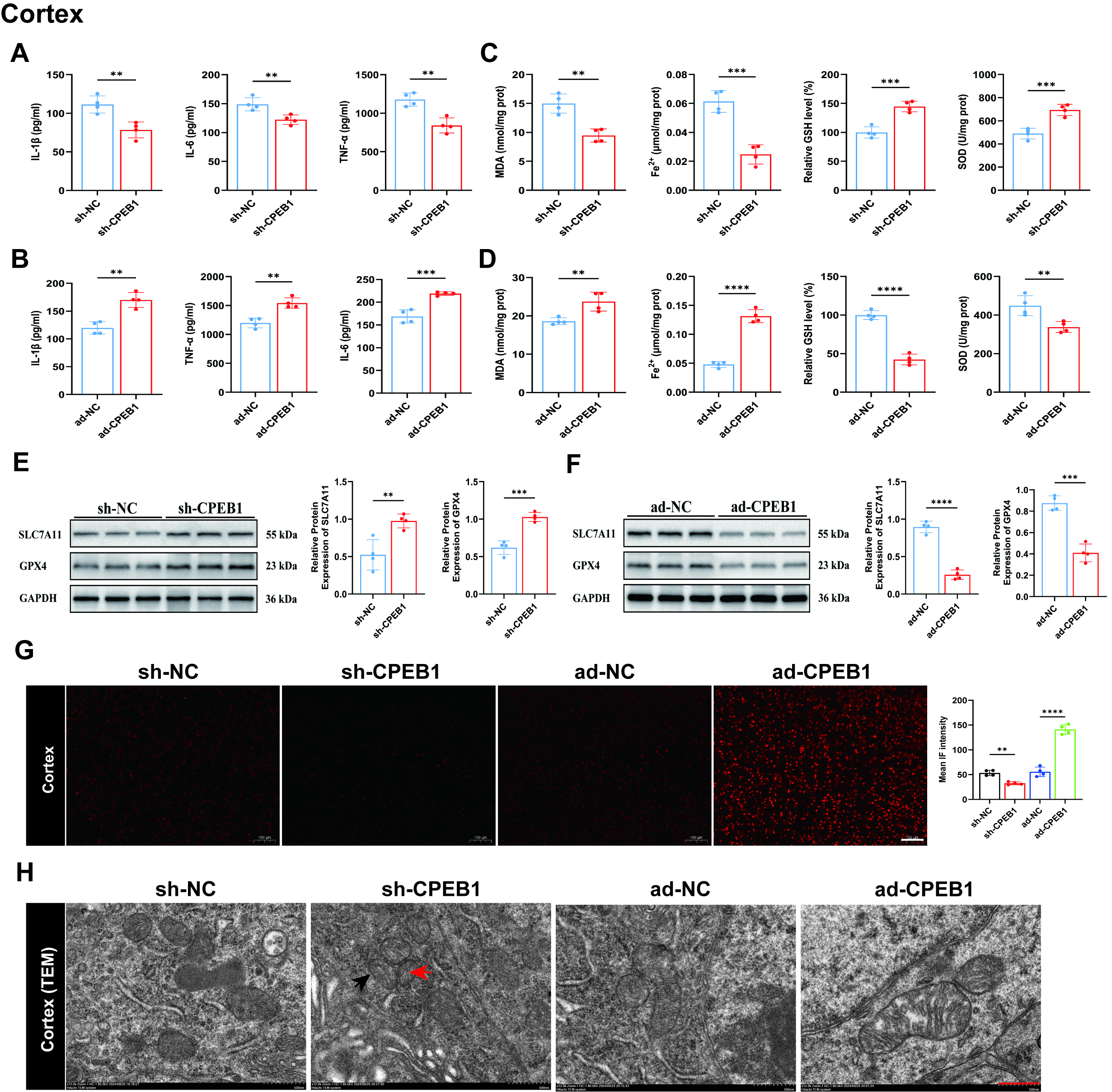

Supplement: Supplementary Figure 6 — CPEB1 regulates neuroinflammation, oxidative stress, and ferroptosis in the cortex of KA-induced epileptic mice. (A) Levels of pro-inflammatory cytokines IL-1β, IL-6, and TNF-α in the cortex of sh-NC and sh-CPEB1 mice following kainic acid (KA)-induced epilepsy. (B) Levels of IL-1β, TNF-α, and IL-6 in the cortex of ad-NC and ad-CPEB1 mice following KA-induced epilepsy. (C) Oxidative stress and ferroptosis-related biochemical indices in the cortex of sh-NC and sh-CPEB1 mice, including malondialdehyde (MDA), Fe²+ content, relative glutathione (GSH) levels, and superoxide dismutase (SOD) activity. (D) Oxidative stress and ferroptosis-related biochemical indices in the cortex of ad-NC and ad-CPEB1 mice. (E) Representative Western blot images and quantitative analysis of SLC7A11 and GPX4 protein expression in the cortex of sh-NC and sh-CPEB1 mice. GAPDH was used as a loading control. (F)Representative Western blot images and quantitative analysis of SLC7A11 and GPX4 protein expression in the cortex of ad-NC and ad-CPEB1 mice. (G) Representative dihydroethidium (DHE) staining images showing reactive oxygen species (ROS) accumulation in the cortex of sh-NC, sh-CPEB1, ad-NC, and ad-CPEB1 mice. Quantification of mean fluorescence intensity is shown on the right. (H) Representative transmission electron microscopy (TEM) images showing mitochondrial ultrastructural changes in cortical neurons. Black arrows indicate mitochondrial membranes, and red arrows indicate mitochondrial cristae, with membrane disruption and cristae loss representing characteristic features of ferroptosis. Data are presented as mean ± SEM. *P < 0.05, **P < 0.01, ***P < 0.001, ****P < 0.0001. [file Image6.tif]

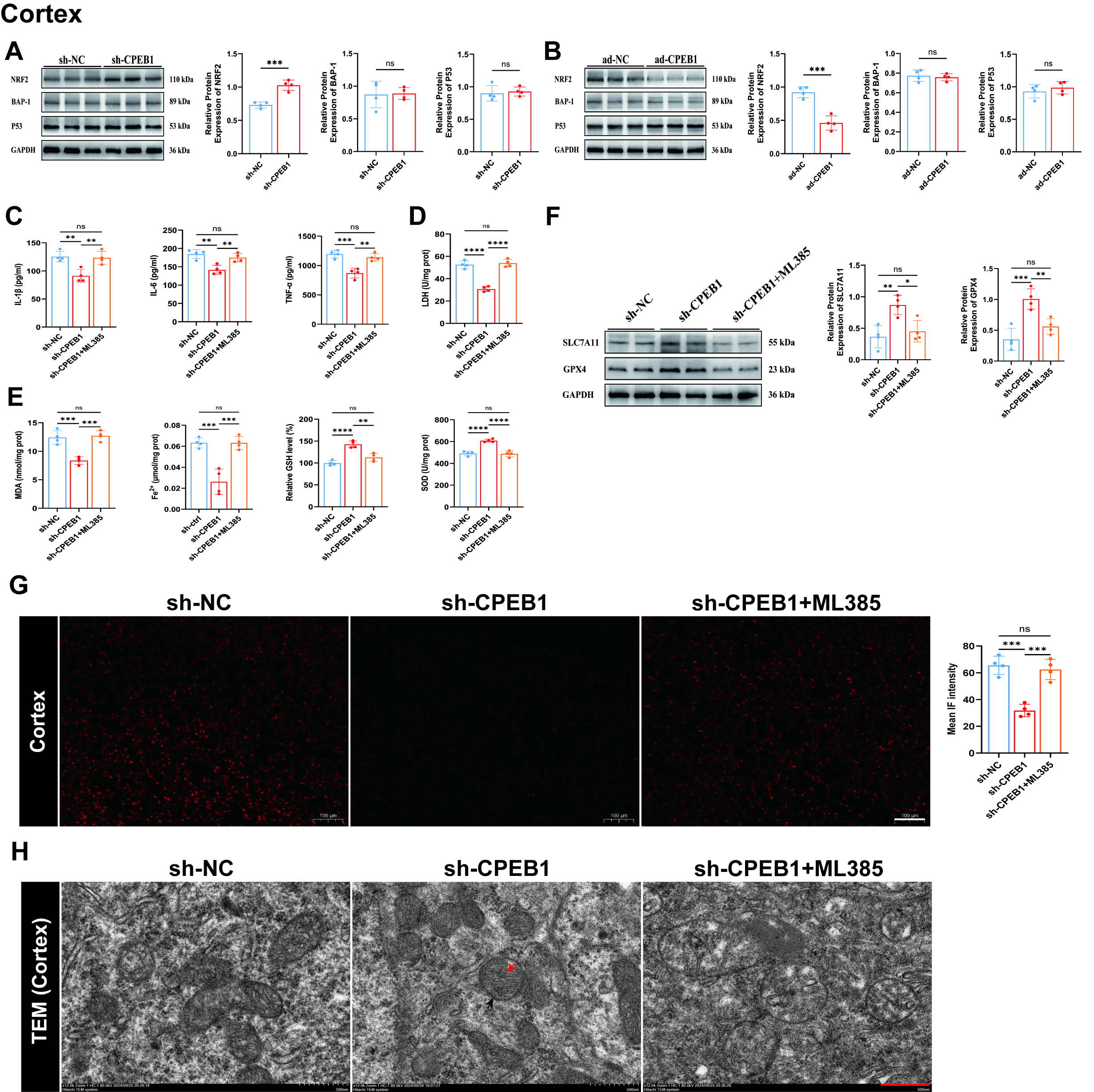

Supplement: Supplementary Figure 7 — CPEB1 regulates NRF2 signaling and ferroptosis in the cortex of KA-induced epileptic mice. (A) Representative Western blot images and quantitative analysis of NRF2, BAP1, and P53 protein expression in the cortex of sh-NC and sh-CPEB1 mice following kainic acid (KA)-induced epilepsy. (B) Representative Western blot images and quantitative analysis of NRF2, BAP1, and P53 protein expression in the cortex of ad-NC and ad-CPEB1 mice following KA-induced epilepsy. (C) Levels of pro-inflammatory cytokines IL-1β, IL-6, and TNF-α in the cortex of sh-NC, sh-CPEB1, and sh-CPEB1 + ML385 mice. (D) Lactate dehydrogenase (LDH) release levels in the cortex of sh-NC, sh-CPEB1, and sh-CPEB1 + ML385 mice. (E) Oxidative stress and ferroptosis-related biochemical indices in the cortex, including malondialdehyde (MDA), Fe²+ content, relative glutathione (GSH) levels, and superoxide dismutase (SOD) activity. (F) Representative Western blot images and quantitative analysis of SLC7A11 and GPX4 protein expression in the cortex of sh-NC, sh-CPEB1, and sh-CPEB1 + ML385 mice. (G) Representative dihydroethidium (DHE) staining images showing reactive oxygen species (ROS) accumulation in the cortex of sh-NC, sh-CPEB1, and sh-CPEB1 + ML385 mice. Quantification of mean fluorescence intensity is shown on the right. (H) Representative transmission electron microscopy (TEM) images showing mitochondrial ultrastructural alterations in cortical neurons. Black arrows indicate mitochondrial membranes, and red arrows indicate mitochondrial cristae. Mitochondrial membrane disruption and cristae loss are characteristic morphological features of ferroptosis. Data are presented as mean ± SEM. ns, not significant; *P < 0.05, **P < 0.01, ***P < 0.001, ****P < 0.0001. [file Image7.tif]

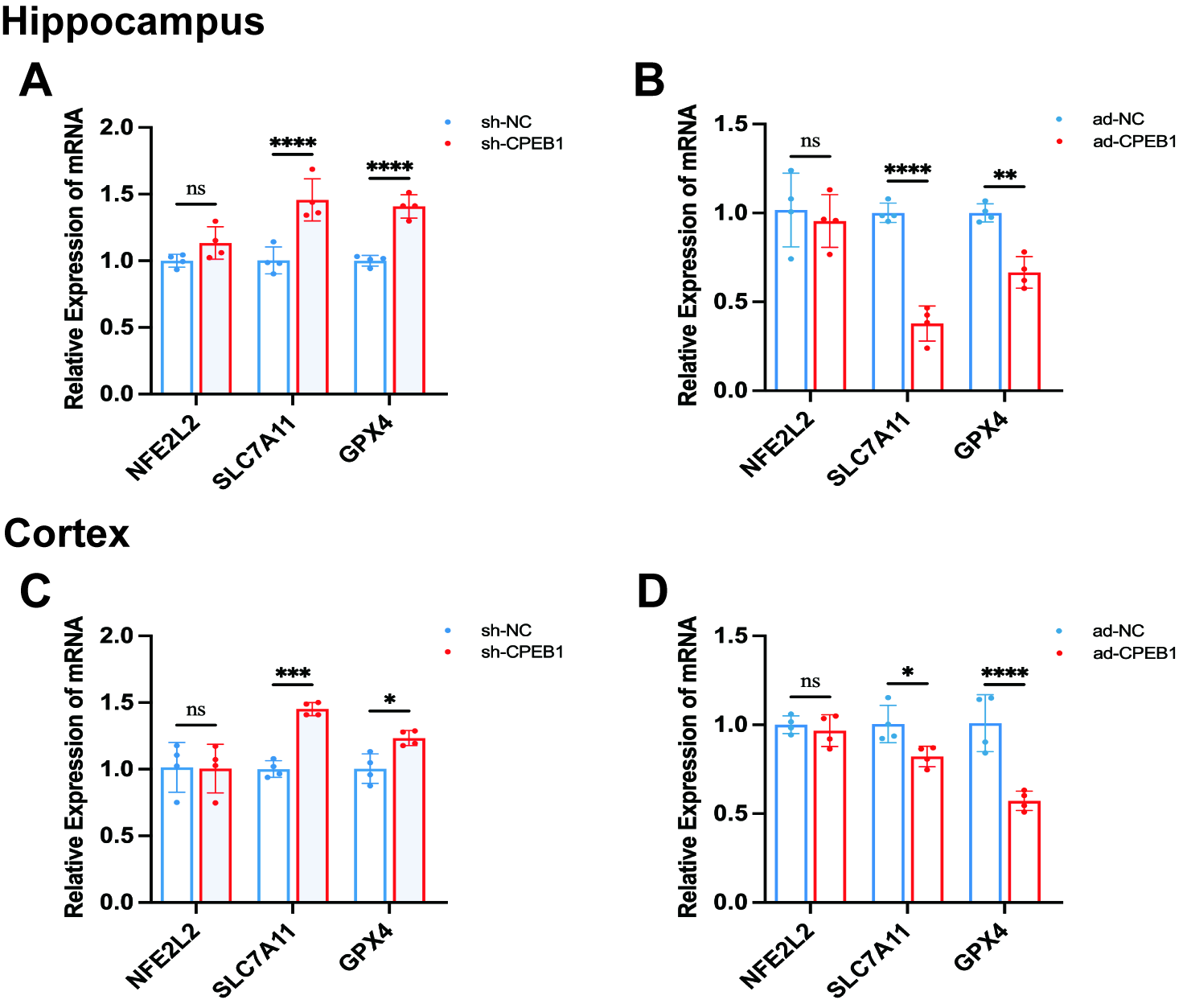

Supplement: Supplementary Figure 8 — Quantification of NFE2L2, SLC7A11, and GPX4 mRNA expression in hippocampal and cortical tissues of CPEB1-manipulated mice. (A, B) Relative mRNA expression levels of NFE2L2 (NRF2), SLC7A11, and GPX4 in hippocampal tissue from sh-CPEB1 and ad-CPEB1 mice compared with their respective controls (sh-NC and ad-NC). (C, D) Corresponding qPCR analysis performed in cortical tissue. Gene expression values were normalized to GAPDH and analyzed using the 2-ΔΔCt method. Data are presented as mean ± SD (n = 4). Statistical significance was determined using one-way ANOVA with Tukey’s test. ns: not significant; *p < 0.05; **p < 0.01; ***p < 0.001; ****p < 0.0001. [file Image8.tif]

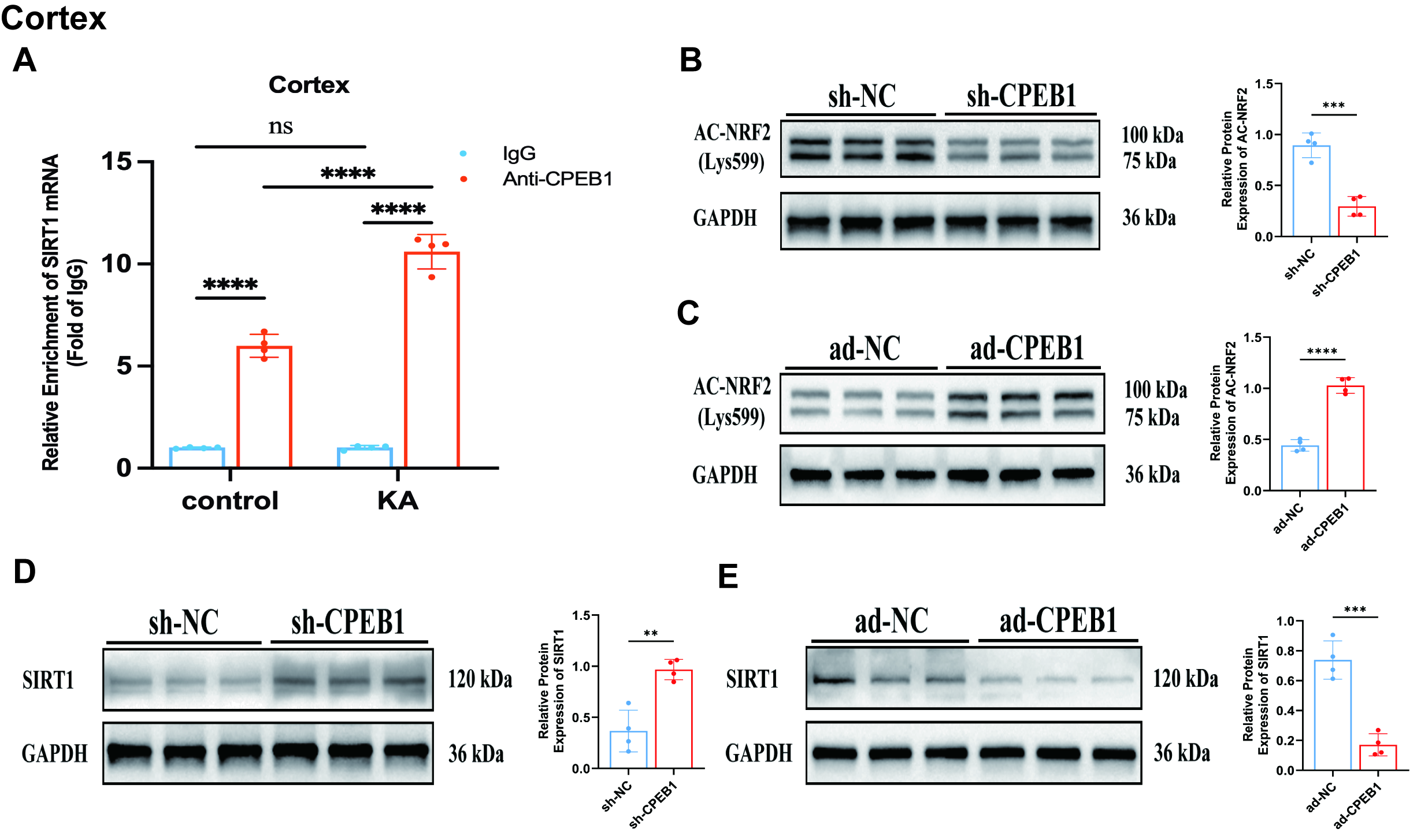

Supplement: Supplementary Figure 9 — CPEB1 regulates SIRT1 expression and NRF2 acetylation in the cortex of KA-induced epileptic mice. (A) RNA immunoprecipitation (RIP) assay showing enrichment of SIRT1 mRNA pulled down by anti-CPEB1 antibody or IgG control in the cortex of control and kainic acid (KA)-treated mice. (B) Representative Western blot images and quantitative analysis of acetylated NRF2 (Ac-NRF2, Lys599) protein levels in the cortex of sh-NC and sh-CPEB1 mice. (C) Representative Western blot images and quantitative analysis of Ac-NRF2 protein levels in the cortex of ad-NC and ad-CPEB1 mice. (D) Representative Western blot images and quantitative analysis of SIRT1 protein expression in the cortex of sh-NC and sh-CPEB1 mice. (E) Representative Western blot images and quantitative analysis of SIRT1 protein expression in the cortex of ad-NC and ad-CPEB1 mice. Data are presented as mean ± SEM. ns, not significant; **P < 0.01, ***P < 0.001, ****P < 0.0001. [file Image9.tif]

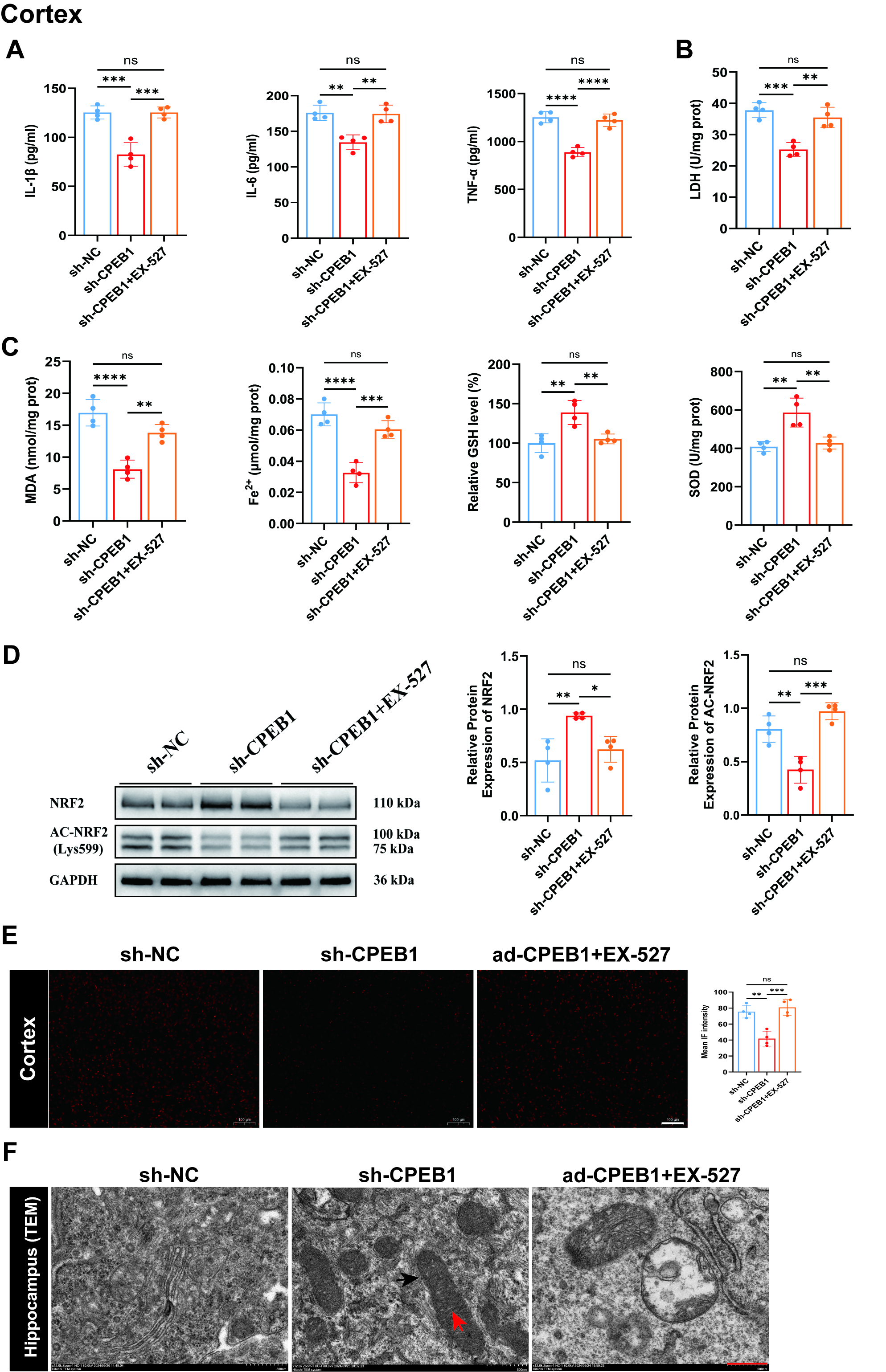

Supplement: Supplementary Figure 10 — Inhibition of SIRT1 reverses the effects of CPEB1 knockdown on inflammation, oxidative stress, and ferroptosis in the cortex. (A) Levels of pro-inflammatory cytokines IL-1β, IL-6, and TNF-α in the cortex of sh-NC, sh-CPEB1, and sh-CPEB1 + EX-527 mice following kainic acid (KA)–induced epilepsy. (B) Lactate dehydrogenase (LDH) release levels in the cortex of sh-NC, sh-CPEB1, and sh-CPEB1 + EX-527 mice. (C) Oxidative stress and ferroptosis-related biochemical indices in the cortex, including malondialdehyde (MDA), Fe²+ content, relative glutathione (GSH) levels, and superoxide dismutase (SOD) activity. (D) Representative Western blot images and quantitative analysis of NRF2 and acetylated NRF2 (Ac-NRF2, Lys599) protein expression in the cortex of sh-NC, sh-CPEB1, and sh-CPEB1 + EX-527 mice. (E) Representative dihydroethidium (DHE) staining images showing reactive oxygen species (ROS) accumulation in the cortex of sh-NC, sh-CPEB1, and sh-CPEB1 + EX-527 mice. Quantification of mean fluorescence intensity is shown on the right. (F) Representative transmission electron microscopy (TEM) images showing mitochondrial ultrastructural alterations. Black arrows indicate mitochondrial membranes, and red arrows indicate mitochondrial cristae. Disruption of mitochondrial membranes and loss of cristae are characteristic morphological features of ferroptosis. Data are presented as mean ± SEM. ns, not significant; *P < 0.05, **P < 0.01, ***P < 0.001, ****P < 0.0001. [file Image10.tif]
