# Supplementary material for: CPEB1 drives ferroptosis–neuroinflammation crosstalk in temporal lobe epilepsy via the SIRT1–NRF2 acetylation axis
Source: Front Immunol. 2026 Mar 13;17:1727784. doi: 10.3389/fimmu.2026.1727784 (PMC13021461; doi:10.3389/fimmu.2026.1727784)
Supplement: Supplementary file 11 [file DataSheet1.docx]

Supplementary Material

# Materials and methods

## Single-cell RNA-seq Data Processing and Quality Control

## The single-cell RNA sequencing (scRNA-seq) dataset GSE190452, derived from hippocampal tissues of TLE patients, was retrieved from the Gene Expression Omnibus (GEO) database. All downstream analyses were performed using the Seurat R package (v4.0) (1). Raw gene expression matrices were first subjected to stringent quality control to remove low-quality cells and potential doublets. Low-quality cells were filtered based on the following criteria: (1) cells with fewer than 500 detected genes (nFeature_RNA > 500); (2) cells with aberrant transcriptional output, defined as total Unique Molecular Identifier (UMI) counts outside the range of 1,000 to 20,000 (1,000 < nCount_RNA < 20,000); and (3) cells demonstrating cellular stress, indicated by a mitochondrial gene proportion greater than 10% (percent.mt < 10%). After filtration, the data were normalized using the NormalizeData function via the "LogNormalize" method with a scale factor of 10,000. The top 2,000 highly variable genes (HVGs) were identified using the FindVariableFeatures function (vst selection method) to drive downstream dimensionality reduction.

## Dimensionality Reduction, Clustering, and Cell Type Annotation

To reduce dataset dimensionality, Principal Component Analysis (PCA) was performed on the scaled data of the identified HVGs. The optimal number of principal components (PCs) for clustering was determined using the ElbowPlot function, and the top 20 PCs were selected for subsequent analysis. Unsupervised cell clustering was conducted using a graph-based approach with the FindNeighbors function followed by FindClusters at a resolution of 0.5. For visualization, non-linear dimensionality reduction was performed using t-Distributed Stochastic Neighbor Embedding (t-SNE). Cell type annotation was performed using a combined automated and manual strategy. The SingleR R package(2) was utilized for automated cell type recognition against the Human Primary Cell Atlas reference dataset. These predictions were further validated manually using canonical marker genes retrieved from the CellMarker database(3) (http://xteam.xbio.top/CellMarker). Differentially expressed genes (DEGs) for each cluster were identified using the FindAllMarkers function with the Wilcoxon rank-sum test (adjusted p-value < 0.05).

## Bulk RNA-seq Integration and Identification of Pathogenic Targets

To identify robust disease-associated targets, we integrated a large-scale bulk RNA-seq dataset (GSE256068) comprising 59 TLE brain tissue samples and 11 normal controls(4). Differential expression analysis between TLE and control groups was performed using the limma package(5) in R. Significant DEGs were defined by a threshold of |log₂Fold Change| > 1 and p-value < 0.05. To screen for key ferroptosis-related drivers in TLE, we performed a multi-dimensional intersection analysis. We intersected the TLE-associated neuron-specific DEGs (identified from scRNA-seq), the bulk tissue DEGs (from GSE256068), and epilepsy-associated genes curated from the GeneCards database (Relevance score > 10). The resulting core gene set was further intersected with the FerrDb database (http://www.zhounan.org/ferrdb/) to pinpoint regulators of ferroptosis, leading to the identification of CPEB1 and SIRT1.

## Functional Enrichment Analysis

To elucidate the biological functions and signaling pathways associated with the identified DEGs, Gene Ontology (GO) and Kyoto Encyclopedia of Genes and Genomes (KEGG) pathway enrichment analyses were conducted using the clusterProfiler R package. The GO analysis covered biological processes (BP), cellular components (CC), and molecular functions (MF). An adjusted p-value (Benjamini-Hochberg correction) of < 0.05 was considered statistically significant.

## mRNA Sequence Retrieval and 3D Structure Prediction

The human SIRT1 mRNA sequence was obtained from the NCBI GenBank database (Accession No. NM_001142498.2). A conserved CPEB1-binding site (CPE core motif: 5’-UUUUUAU-3’) was identified within the 3’ untranslated region (3’ UTR). To construct a 3D structural model of this region, an 80-nucleotide fragment encompassing the CPE motif and its flanking sequences (corresponding to positions ~3800–3880 bp, with T replaced by U) was extracted. The atomic-resolution RNA 3D model was generated using the RNAComposer algorithm. This method, based on the Machine Translation System (MTS) principle, first predicts the optimal secondary structure (minimum free energy state) via RNAfold, then maps the secondary topology to 3D structural elements, followed by structure refinement using an all-atom potential-based energy minimization algorithm to yield a high-resolution PDB file.

## Protein Structure Preparation

The 3D structure of the RNA recognition motif (RRM) domain of human CPEB1 was retrieved from the RCSB PDB database (PDB ID: 2MKH). The first model (Model 1) from this NMR ensemble was used as the receptor, covering residues 219–434. Prior to docking, solvent molecules and non-standard heteroatoms were removed.

## Molecular Docking and Interface Interaction Analysis

The protein-RNA complex was constructed using the HDOCK algorithm. HDOCK applies a hybrid docking strategy combining template-based modeling and FFT-based ab initio search, allowing for conformational flexibility of both receptor and ligand during docking. The system generates the top 10 models based on the proprietary ITScorePP scoring function and RMSD-based clustering. The model ranked first (Rank 1) based on the lowest energy was selected for subsequent binding mode analysis. The Arpeggio algorithm was used to calculate non-covalent interactions at the complex interface, with a cutoff value set to 4.0 Å. Interactions were classified according to geometric and chemical criteria, including hydrogen bonds, ionic interactions, aromatic stacking, and hydrophobic contacts. All structural visualizations were generated using PyMOL (v2.5), and interaction types were annotated following the standard Arpeggio scheme.

## Reactive oxygen species (ROS) determination

To assess oxidative stress alterations in epilepsy models, dihydroethidium (DHE) staining was performed to detect reactive oxygen species (ROS) production in brain tissues. Frozen brain sections were incubated with 10 μmol/L DHE staining solution at 37°C for 30 minutes in the dark. Following incubation, sections were rapidly rinsed with PBS to remove excess dye. Fluorescence images were then acquired using a Nikon fluorescence microscope (Nikon, Japan), with DHE oxidation products emitting red fluorescence as an indicator of intracellular ROS levels. This experiment was designed to determine whether CPEB1 regulation promotes neuronal ferroptosis in epilepsy by modulating ROS accumulation.

## Measurement of Malondialdehyde (MDA)

To evaluate lipid peroxidation in brain tissues, malondialdehyde (MDA) levels were measured using a commercial assay kit (Beyotime, China; Catalog No.: S0131S), following the manufacturer’s instructions. Briefly, brain tissue homogenates were prepared and reacted with thiobarbituric acid (TBA) to generate MDA–TBA adducts, which were subsequently quantified spectrophotometrically at a defined wavelength. MDA levels were expressed as nmol/mg protein, reflecting the extent of lipid peroxidation in cellular membranes. As a classical biochemical marker of ferroptosis, MDA served as a key index for assessing the contribution of CPEB1 regulation to epilepsy-associated oxidative stress injury.

## Glutathione (GSH) Content Measurement

To assess alterations in the antioxidant capacity of brain tissues, glutathione (GSH) levels were measured using a commercial GSH/oxidized glutathione (GSSG) assay kit (Beyotime, China; Catalog No.: S0053), following the manufacturer’s instructions. Briefly, tissue homogenates were prepared, and GSH was subjected to an enzymatic reaction with a specific substrate to produce a chromogenic product, which was subsequently quantified spectrophotometrically at a defined wavelength. GSH levels in each experimental group were normalized to those of the control group and expressed as relative percentages. As a critical antioxidant molecule in ferroptosis regulation, GSH was used as a key indicator to evaluate the effects of CPEB1 on neuronal antioxidant capacity in the epilepsy model.

## Ferrous Ion (Fe²⁺) Measurement

To evaluate iron accumulation during ferroptosis, ferrous ion (Fe²⁺) concentrations in brain tissues were measured using a commercial assay kit (Solarbio, China; Catalog No.: BC5415), in strict accordance with the manufacturer’s instructions. Briefly, brain tissues were homogenized, centrifuged, and the resulting supernatant was incubated with a chromogenic reagent to generate a stable colored complex, which was quantified spectrophotometrically at a defined wavelength. Fe²⁺ content was expressed as μmol/mg protein. As a central driver of lipid peroxidation chain reactions, Fe²⁺ accumulation is a key hallmark of ferroptosis. This experiment was conducted to further determine whether CPEB1 contributes to neuronal ferroptosis in epilepsy through the regulation of iron metabolism.

## Superoxide Dismutase (SOD) Activity Assay

To assess the function of the antioxidant enzyme system in brain tissues, superoxide dismutase (SOD) activity was measured using a commercial assay kit (Solarbio, China; Catalog No.: BC0170), following the manufacturer’s instructions. Briefly, brain tissue homogenates were prepared and incubated with reaction substrates, and the degree of inhibition of superoxide anion radical generation was used as an indirect indicator of SOD activity. The reaction products were subsequently quantified colorimetrically at a defined wavelength, and SOD activity was expressed as U/mg protein. As a critical intracellular antioxidant enzyme, SOD scavenges superoxide radicals and mitigates oxidative stress. Changes in its activity were therefore used to evaluate whether CPEB1 contributes to epilepsy-associated ferroptosis by modulating the antioxidant defense system.

## Lactate dehydrogenase (LDH) Activity Assay

To assess the extent of cellular injury, lactate dehydrogenase (LDH) activity in brain tissues was measured using a commercial assay kit (Solarbio, China; Catalog No.: BC0685), following the manufacturer’s instructions. Briefly, brain tissues were homogenized and centrifuged, and the resulting supernatant was incubated with reaction substrates to catalyze the conversion of lactate to pyruvate, accompanied by the generation of NADH. The reaction products were then quantified colorimetrically at a defined wavelength to determine enzymatic activity. LDH activity was expressed as U/mg protein. As a classical marker of disrupted cell membrane integrity, elevated LDH activity is indicative of increased cellular injury or death. This assay was employed to further evaluate whether CPEB1 promotes neuronal injury in epilepsy models through ferroptosis-related pathological mechanisms.

## Enzyme-Linked Immunosorbent Assay (ELISA)

After dissection, cortical and hippocampal tissues were immediately isolated from mouse brains under ice-bath conditions. The tissues were homogenized in PBS buffer and centrifuged at 5000 rpm for 15 minutes at 4°C, after which the supernatants were collected. The concentrations of interleukin-1β (IL-1β), interleukin-6 (IL-6), and tumor necrosis factor-α (TNF-α) in cortical and hippocampal tissues were quantified using commercial ELISA kits (Solarbio, Beijing, China; Catalog Nos.: SEKM-0002, SEKM-0007, SEKM-0034), strictly according to the manufacturer’s instructions(6).

**References**

1. Hu Y, Wan S, Luo Y, Li Y, Wu T, Deng W, et al. Benchmarking algorithms for single-cell multi-omics prediction and integration. Nat Methods. 2024;21(11):2182–94.

2. Huang Q, Liu Y, Du Y, Garmire LX. Evaluation of Cell Type Annotation R Packages on Single-cell RNA-seq Data. Genomics Proteomics Bioinformatics. 2021;19(2):267–81.

3. Hu C, Li T, Xu Y, Zhang X, Li F, Bai J, et al. CellMarker 2.0: an updated database of manually curated cell markers in human/mouse and web tools based on scRNA-seq data. Nucleic Acids Res. 2023;51(D1):D870–d6.

4. François L, Romagnolo A, Luinenburg M, Anink J, Godard P, Rajman M, et al. Identification of gene regulatory networks affected across drug-resistant epilepsies. Nature communications. 2024;15(1):2180.

5. Baker BH, Sathyanarayana S, Szpiro AA, MacDonald JW, Paquette AG. RNAseqCovarImpute: a multiple imputation procedure that outperforms complete case and single imputation differential expression analysis. Genome Biol. 2024;25(1):236.

6. Lu Y, Lin M, Ou S, Sun L, Qian K, Kuang H, et al. Astragalus polysaccharides ameliorate epileptogenesis, cognitive impairment, and neuroinflammation in a pentylenetetrazole-induced kindling mouse model. Front Pharmacol. 2024;15:1336122.
